# Atypical odontalgia and trigeminal neuralgia: psychological, behavioural and psychopharmacologic approach – an overview of the pathologies related to the challenging differential diagnosis in orofacial pain

**DOI:** 10.12688/f1000research.51845.2

**Published:** 2021-10-18

**Authors:** Riccardo Tizzoni, Marta Tizzoni, Carlo Alfredo Clerici

**Affiliations:** 1 Independent Reasercher, 20122 Milano, Italy; 2 Department of Oncology and Haematology-Oncology,, University of Milano, 20122 Milano, Italy; 3 Clinical Psychology Unit, Fondazione IRCCS, Istituto Nazionale dei Tumori, 20133 Milano, Italy

**Keywords:** atypical odontalgia, trigeminal neuralgia, case report, psychiatric disorders, orofacial pain

## Abstract

Orofacial pain represents a challenge for dentists, especially if with a non-odontogenic basis. Orofacial neuropathic pain is chronic, arduous to localize and develops without obvious pathology. Comorbid psychiatric disorders, such as anxiety and depression, coexist and negatively affect the condition. This article presents one case of atypical odontalgia and one of trigeminal neuralgia treated with psychological and psychopharmacologic tailored and adapted therapies, after conventional medications had failed.

In addition, an overview of the pathologies related to the challenging differential diagnosis in orofacial pain is given, since current data are insufficient.

A 68-year-old male complained of chronic throbbing, burning pain in a maxillary tooth, worsening upon digital pressure. Symptoms did not abate after conventional amitriptyline therapy; psychological intervention and antianxiety drug were supplemented and antidepressant agent dosage incremented; the patient revealed improvement and satisfaction with the multidisciplinary approach to his pathology. A 72-year-old male lamented chronic stabbing, intermittent, sharp, shooting and electric shock-like pain in an upper tooth, radiating and following the distribution of the trigeminal nerve. Pain did not recur after psychological intervention and a prescription of antidepressant and antianxiety agents, while conventional carbamazepine therapy had not been sufficient to control pain. Due to concern with comorbid psychiatric disorders, we adopted a patient-centered, tailored and balanced therapy, favourably changing the clinical outcome.

Comorbid psychiatric disorders have a negative impact on orofacial pain and dentists should consider adopting tailored therapies, such as psychological counselling and behavioural and psychopharmacologic strategies, besides conventional treatments. They also need to be familiar with the signs and symptoms of orofacial pain, recollecting a comprehensive view of the pathologies concerning the differential diagnosis. A prompt diagnosis prevents pain chronicity, avoiding an increase in complexity and a shift to orofacial neuropathic pain and legal claims.

## Introduction

In clinical dental practice there are difficult situations to be managed by the practitioner, but the most challenging and gratifying are those related to the diagnosis and treatment of pain, especially if with a non-odontogenic basis. ^1^

In the orofacial region, aesthetic, biological, emotional and relational importance and psychological sphere prominence have to be considered. ^2^

Pre-existing pre-treatment, especially long-lasting, inflammatory pain seems to have paramount importance and might represent a key factor to evolve into orofacial neuropathic pain in the same area. ^3^ Therefore, it is advisable to avoid an increase in complexity and a shift to chronic pain, conditions which may dispose to psychological distress ^2^ and may magnify the degree of pain and its characteristics.

The patient may become deprived of confidence and hope due to diagnostic procrastination, irreversible dental treatments and lack of knowledge and may experience frustration up to clinical depression. ^4^^,^^5^

Prevention, early diagnosis, and treatment of inflammatory pain may thus have utility in avoiding the development of neuropathic pain ^3^ and consequent anxiety. ^2^^,^^6^

Depression is a mood disorder characterized by persistent sadness associated with other symptoms. The four most common types of depression are major depression (a bad temper associated with reduction in psychological vitality, or even inability to experience pleasure, and a decrease of other physiological functions, such as sleep), persistent depressive disorder (a form of depression which has lasted for at least two years without reaching the magnitude of major depression), bipolar disorder (a form characterized by episodes of depression, alternating with intervals of unusually high energy or vitality) and seasonal affective disorder (a mental condition which characteristically arises in autumn and winter as a consequence of alterations in the body’s natural daily rhythms, in the eyes’ sensitivity to light, or in changes in serotonin and melatonin chemical messages).

Two other depression types, unique to women, are those influenced by reproductive hormones: perinatal depression and premenstrual dysphoric disorder. ^7^

Anxiety is a mental state that arises spontaneously rather than through conscious effort and is characterized and often accompanied by stress, worried thoughts and physical changes. ^8^

Based on available psychometric evidence, the Beck depression inventory – version two (BDI-II) can be viewed as a cost-effective, reliable tool to measure depression intensity of a patient, with broad applicability for research and clinical practice. It can easily discriminate between depressed and non-depressed subjects. ^9^

It is also of paramount importance to understand the intensity of patient anxiety since this allows appropriate management. However, anxiety level is not easily measured. There are different methods accessible to dentists to measure patient dental anxiety; for example, the modified dental anxiety scale (MDAS). ^10^

There are also many strategies available to the dental team to safely provide comprehensive care to quell patient anxiety and depression.

When patients afflicted with orofacial pain and associated comorbid psychiatric disorders cannot be treated by conventional therapies, reassuring them by providing an explanation and comforting professional empathy, counselling and psychological therapy should be considered, and well-tolerated and effective antidepressants and antianxiety drugs should be prescribed.

In many cases of orofacial pain, due to the absence of radiographic signs and clinical symptoms of endodontic or restorative dentistry related pathologies, or other identifiable causes, ^11^^,^^12^ the dental practitioner should be very well trained and also aware of the difficult differential diagnostic process (a mental activity and computing, following logical methods, which utilizes the combination of knowledge and reasoning about current symptoms, medical history, results from physical and possibly laboratory or other examinations and discriminates amongst different diseases which partially share signs or symptoms, specific signs or symptoms of the pathology intended to be diagnosed).

The differential diagnostic process and treatment are extremely important while dealing with these pathologies, to avoid diagnostic delays, useless and superfluous dental treatments ^4^ and risks of legal claims.

Amongst other orofacial pain, atypical odontalgia (AO), also termed persistent dentoalveolar pain disorder, ^4^ and trigeminal neuralgia (TN) are the main diagnosed neuropathic pains. ^2^

AO can affect up to 6% of patients after they have undergone endodontic therapies ^4^^,^^13^ and usually leads to tooth extraction without recovery of the pain. ^11^

There is heterogeneity in the classification proposed in the literature but, in accordance with the third edition of the international classification of headache disorders (ICHD-3), this condition is now classified as a subtype of persistent idiopathic facial pain or, due to occasional presence of traumatic trigger, may also be considered a subdivision of post-traumatic painful trigeminal neuropathy. ^14^

Patients affected by AO describe the pain as localized to the tooth, or to the tooth surrounding bone, generally continuous, with periods of relief, aching, dull, throbbing, sometimes sharp, with a mild to moderate intensity. Paraesthesia or dysesthesia may be detected during examination. ^1^ Sometimes touching of the area may represent an aggravating factor.

The pathophysiological mechanisms to justify the onset and persistence of the condition are not fully elucidated, ^4^ but the most endorsed hypothesis is that of a neuropathic origin, assuming that injuries to teeth and/or periodontal tissues may modify health status, leading to alteration in the periodontal nerve plexus and resulting in peripheral sensitization. ^15^

In the pathophysiological mechanisms of AO, comorbid psychiatric disorders should also be taken into account. In a recent study, ^16^ 46.2% of patients with AO showed comorbid psychiatric disorders. Of those patients, 15.4% showed depressive disorders and 10.1% showed anxiety disorders. Serious mental disorders like bipolar disorder and schizophrenia were present only in 3.0% and 1.8% of the cases, respectively. Thus, pain might have a significant emotional basis, besides the previously discussed sensory one. ^16^ Table 1 reports the diagnostic criteria for the diagnosis of AO. ^14^ TN is reportedly the most frequent condition (82.1%) amongst patients with neuropathic pain. ^2^

**Table 1.** Diagnostic criteria for the diagnosis of Atypical Odontalgia (ICHD-3 classification ^14^).

Atypical Odontalgia		The term has been applied to a continuous pain in one or more teeth or in a tooth socket after extraction, in the absence of any usual dental cause.
		This is thought to be a subtype of Persistent idiopathic facial pain although it is more localized, the mean age at onset is younger and genders are more balanced.
		Based on the history of trauma, atypical odontalgia may also be a subform of Painful post-traumatic trigeminal neuropathy. These subtypes/forms, if they exist, have not sufficiently studied to propose diagnostic criteria.
Persistent idiopathic facial pain (PIFP)	A	Facial and/or oral pain fulfilling criteria B and C
	B	Recurring daily for >2 hours/day for >3 months
	C	Pain has both of the following characteristics: 1. poorly localized, and not following the distribution of a peripheral nerve; 2. dull, aching or nagging quality
	D	Clinical neurological examination is normal
	E	A dental cause has been excluded by appropriate investigations
	F	Not better accounted for by another ICHD-3 diagnosis

TN is characterized by paroxysmal, sharp, severe unilateral pain in the distribution of the trigeminal nerve, although patients may experience a variety of symptoms that simulate pain of odontogenic origin. ^1^

Trigger areas around the nose and the mouth characterize the condition, provoking the sudden onset of the pain which can lasts seconds to minutes, giving relief to patients with pain free intervals. ^17^

From a pathophysiological standpoint, as the most supported theory, it is assumed that tumour or vascular compression may lead to partial and focal nerve demyelination and consequent abnormal transmission and processing of impulses along the trigeminal nerve. ^1^^,^^18^^,^^19^ More specifically, as regards the pathophysiology of paroxysmal pain, vascular compression is the usual cause of demyelination at the site just before the nerve enters the pons, and multiple sclerosis is the typical cause at the site just after entry into the pons. ^20^

Recent diagnostic criteria distinguish TN as “classical”, related to neurovascular compression producing morphological changes on the trigeminal root, “secondary” to a major neurological disease or “idiopathic” with unknown etiology. Genetic factors may also play a role in the pathophysiology of idiopathic TN. ^20^^,^^21^ Chronic irritation or trauma have also been thought to be involved in the origin of TN. ^1^

In the development of TN, magnetic resonance imaging (MRI) supported the most recent evidence of superior cerebellar artery aneurysms ^22^ and venous compression, ^23^ but also revealed benign or malignant lesions and plaques of multiple sclerosis. ^22^ Moreover, TN affects 1% of patients with multiple sclerosis and 2% to 8% of patients with TN are affected by multiple sclerosis. ^18^ Pain characteristics of TN often have unfavourable effects on the daily life of patients. Patients are affected by disruptive pain with a consequent poor quality of life and significantly reduced working performance. ^24^ For these reasons, it is also important to evaluate the psychological aspects of patients affected by TN. Moreover, up to 50% of patients also experience concomitant continuous pain. In a clinical and neuroimaging study was found that in patients with TN, concomitant continuous pain was associated with trigeminal nerve atrophy. In these cases, this type of pain is probably related to axonal loss and abnormal activity in denervated trigeminal second-order neurons. ^25^

Table 2 reports the diagnostic criteria for the diagnosis of TN. ^14^ In this article, due to the above mentioned comorbid psychiatric disorders, one case of AO and one case of TN are presented and discussed taking into consideration patient-centered, tailored and adapted therapies, compared to conventional treatment: strategies for psychological suffering managing were introduced, combining psychological counselling and cognitive behavioural therapies with antidepressants and antianxiety drugs while managing patients affected with these diseases, after conventional therapy had exhibited limited efficacy.

**Table 2.** Diagnostic criteria for the diagnosis of Trigeminal Neuralgia (ICHD-3 classification ^14^).

Trigeminal neuralgia	A	Recurrent paroxysms of unilateral facial pain in the distribution(s) of one or more divisions of the trigeminal nerve, with no radiation beyond, and fulfilling criteria B and C
	B	Pain has all of the following characteristics: 1) Lasting from fraction of a second to two minutes 2) Severe intensity 3) Electric shock-like, shooting, stabbing or sharp in quality
	C	Precipitated by innocuous stimuli within the affected trigeminal distribution
	D	Not better accounted for by another ICHD-3 diagnosis
		Notes: 1) In a few patients, pain may radiate to another division, but it remains within the trigeminal dermatomes. 2) Duration can change over time, with paroxysms becoming more prolonged. A minority of patients will report attacks predominantly lasting for >2 minutes. 3) Pain may become more severe over time 4) Some attacks may be, or appear to be, spontaneous, but there must be a history or finding of pain provoked by innocuous stimuli to meet this criterion. Ideally, the examining clinician should attempt to confirm the history by replicating the triggering phenomenon. However, this may not always be possible because of the patient’s refusal, awkward anatomical location of the trigger and/or other factors.

There are several available data on single form of orofacial pain but there is still a lack of knowledge and a lack of up to date available data summarizing, or fully describing, different pain arising from the regions of the face and mouth, which would enable dental practitioners to become familiar with the signs and symptoms of orofacial pain, especially if related to non-odontogenic pain. ^4^^,^^26^

On the bases of these reasons the two case reports acted as stimuli to accomplish also an overview of the pathologies concerning the difficult and challenging differential diagnosis in orofacial pain, which might be a helpful tool for the dental practitioner to broaden the clinical view and bear in mind more information during practice: in fact, a prompt diagnosis prevents pain chronicity, avoiding an increase in complexity and a shift to orofacial neuropathic pain and legal claims.

## Case presentation

### Atypical odontalgia

*Clinical presentation and history*

A Caucasian 68-year-old Italian attorney male was referred to our private practice with the chief complaint of moderate pain in the site of the second maxillary left premolar. The patient complained of a perpetuated period of throbbing or burning pain in the tooth or in the alveolar process, also characterized by a tingling sensation upon digital pressure with a troublesome feeling on his prosthetic zirconia crown. The pain was described as chronic but was absent during sleep, with pain-free intervals during the day. The pain had not been susceptible to non-steroidal anti-inflammatory drugs for six months.

Patient clinical history did not present relevant findings, nor familiar pathologies were referred by the patient. In an addition, an anamnestic psychiatric consultation was scheduled: the patient reported a marked lack of concentration during working hours and considerable impact on his personal and social life. Additionally, the patient displayed anxiety and irritability, especially relating to the difficulties of the diagnostic process. As a consequence of the condition, significant symptoms of depression were referred by the patient.

The tooth had undergone uneventful root canal therapy many years before, a big cast post had been inserted and a gold alloy crown manufactured. The pain had been persisting for about six months, since a dentist had insisted on removing the old gold alloy crown from the tooth to make a new aesthetic zirconia crown.

*Patient assessment*

A comprehensive analysis of the mucosae and gingivae was carried out in quadrants two and three: the neighbouring teeth showed normal responses as a result of testing for vitality with cold; the occlusion was checked and normal and balanced occlusal points were found, and the contact points of the crowns in the area were checked too, to exclude food impaction coexistence. Percussion of the teeth or intra-oral palpation of the above-mentioned quadrant did not provoke pain, with the exception of the second maxillary left premolar.

We also evaluated the function and possible symptoms of the temporomandibular joints; results were within the normal range of motion and without pain. A periapical X-ray of the second maxillary left premolar, and of the neighbouring teeth, was also taken and showed normal tooth and surrounding bone structure, with no signs of pathology. The neuropathic origin of the pain was excluded through neurophysiological tests (trigeminal reflexes) evaluating trigeminal afferents integrity. We based our diagnostic method on the anamnesis, on the comprehensive physical examination, on the X-ray examination, and on the specific anamnestic psychiatric consultation.

Pain intensity was investigated using the short-form McGill pain questionnaire (SF_MPQ). ^27^

*Diagnosis and therapeutic intervention*

Since the patient had been experiencing symptoms for several months, he insisted on having an appointment for tooth extraction, despite our clinical advice. After the patient had signed a specific informed consent form for tooth extraction, and even though painful micro-fractures of the root were thought to be possible due to a big post inserted within the root canal of the tooth for restorative purposes, we reluctantly extracted the tooth and inserted an immediate loaded implant. After topical analgesia had been applied on the vestibular and palatal aspects of the gingivae (Lidocaine 15% spray), local analgesia injection was administered in the vestibular and palatal aspects of the gingivae in the area of the tooth (Mepicain 2%, 1,8 ml, 1:100.000 adrenaline).

After a five-minute period, to allow analgesia onset, a periosteal elevator was used to cut the gingival periodontal fibers and subsequently a forceps was used to gently luxate and ultimately extract the tooth. Since an immediate loaded implant was planned to replace the extracted tooth and for the purpose to obtain primary implant stability, particular attention was paid not to damage the alveolar bone during the extraction. Successively, an osteotomy for implant placement was performed according to the manufacturer’s instruction. The implant (Biomet 3i, 15 mm length x 4mm diameter) got a primary implant stability of 25 Ncm (Newton-centimeter).

After implant insertion, it was not necessary to suture the wound.

After the insertion of the implant, a temporary titanium abutment was selected and a temporary resin crown manufactured. The interpoximal contact points were checked and the temporary crown was cemented on the abutment out of the occlusion to allow proper osseointegration processes. ^28^

Post-operatively, amoxicillin and clavulanic acid (875+125 mg) was prescribed twice a day for 8 days. Chlorhexidine mouth wash 0,20% was recommended three times a day for 15 days. Non-steroidal anti-inflammatory drugs were also prescribed, twice a day on a full stomach (Sodic diclofenac 25 mg) for three days.

For the final prosthetic phases, after a conventional six-month period of healing to allow osseointegration to occur, a superior impression with an open tray and a polyvinyl siloxane material was taken for the replication of the precise position of the implant (rotation, depth in the soft and hard tissues and angulation) relatively to the other oral structures (neighbouring teeth and gingiva).

An alginate impression of the inferior arch was also taken and the bite registered with a polyvinyl siloxane material. The color of the tooth was assessed and a clear lab prescription written. In the lab an upper and lower stone models were obtained and a proper titanium abutment was selected and parallelized for proper crown insertion.

The stone models were then scanned to acquire 3D models to mill a CAD/CAM metal framework of the crown. Afterwards, the abutment and the metal framework were directly checked in the mouth of the patient to evaluate the marginal and internal fit and were sent back to the lab for the final phase of ceramic shaping. During the next dental chair appointment, 8 months after implant insertion, it was possible to screw the titanium abutment and cement the metal-ceramic crown to prosthetically rehabilitate the dental implant of the upper jaw. ^29^

After tooth extraction and implant placement, the patient was strictly followed-up with weekly visits for four weeks and the pain did not remit. Tooth extraction was an unfortunate but an important and discriminating fact, ascertaining that the tooth was not the cause of the pain. In addition, due to the absence of any noticeable odontogenic aetiology and based on the psychological suffering reported by the patient and on the clinical and radiographical findings, the pain was deemed to be enigmatic in origin and perplexing and specifically AO was diagnosed.

The prognostic characteristic of this pathology is generally thought to be a treatment-resistant condition, ^30^ but a multi-disciplinary approach to treatment can lead to a positive outcome. Since the pain (SF-MPQ: score 2) had not remitted after a six-month-period of non-steroidal anti-inflammatory drugs, nor after tooth extraction, we settled for a more patient-centered approach and a combination therapy consisting of psychological counselling, behavioural and pharmacological intervention was prescribed. According to the literature and according to the previous hypothesis of neurogenic pain and specifically of AO, tricyclic antidepressant amitriptyline was prescribed. Nevertheless, symptoms did not abate (SF-MPQ: score 2) after three weeks of increasing doses of amitriptyline (starting dose: 25 mg in the evening for one week; 25 mg in the morning and in the evening during the second week; 25 mg in the morning and 50 mg in the evening during the third week) up to 75 mg per day. Thus, psychological intervention was added (i.e. psychological counselling and cognitive behavioural therapies, based on one session per week with a psychotherapist); also an increment of 25 mg of amitriptyline per week, up to 150 mg per day for six months (then gradually reducing 50 mg per week, until suspension was achieved within three weeks) was prescribed and five drops of clonazepam in the evening for one month (then gradually reducing to three drops for one week and then suspended) were added.

The rationale for the changes in our professional intervention were based on the medical and psychological history, from which we assumed we were facing a case of AO associated with comorbid psychiatric disorders.

*Follow-up and outcomes*

The patient’s psychological sphere difficulties had been revealed by his medical history and upon this basis we decided on psychological counselling and behavioural support and tailored antidepressant and anxiolytic therapy, besides conventional therapy.

The patient reported satisfaction at each follow-up visit with the previously prescribed treatment, which also resulted in a rewarding and gratifying result for the dental team.

Fortunately, dental extraction was not a precipitating event and the clinical case was resolved, from a prosthetic standpoint, with the aid of an implant-supported rehabilitation.

The rehabilitation phases lasted eight months, from first-stage implant insertion surgery to the delivery of the implant-supported ceramic crown. The patient was followed up every 15 days by the dentist and the psychiatrist and was asked about pain intensity (SF-MPQ: score from 1 to 0) and characteristics and psychological conditions. After six months, since he reported decisive improvements in symptoms (SF-MPQ: score 0) and psychological suffering, we prescribed the patient a gradually reduced regimen of antidepressant therapy until suspension. The patient has been pain-free since then. The patient is now in a two-month follow-up programme with the psychiatrist and in a six-month recall programme for dental hygiene.

Psychiatrist and dentist visits were interspersed with phone calls, or with e-mails, or with phone text messages to accomplish a comprehensive check of pharmacotherapy adherence and tolerability. As a consequence, pain, symptoms and psychological conditions were also assessed.

We report no adverse or unanticipated event with regards to the described clinical case.

The diagnosis and treatment of this clinical case was challenging and difficult, even though gratifying, from a differential diagnostic process point of view, especially because the pain had a non-odontogenic basis.

Psychiatric counselling and cognitive behavioural therapies, along with a specific psychopharmacologic approach, are effective treatments for patients suffering acute anxiety, distress and depression while experiencing neuropathic pain. It offers several advantages: the patient may become more compliant and even major pain can be kept under control, with a reduced duration of the symptomatology. The dental office should have a good professional relationship with a specialist psychiatrist.

### Trigeminal neuralgia

*Clinical presentation and history*

A Caucasian 72-year-old Italian engineer male came to our private practice with a six-month history of pain of variable intensity from moderate to severe, in the molar region of the right maxillary quadrant, radiating distant from the tooth area to the ipsilateral region and following the distribution of the branches of the trigeminal nerve. He reported a variety of symptoms and pain characteristics similar to odontogenic pain, which he insisted as originating from the second upper right molar, then radiating to the ipsilateral region.

The patient was eventually able to define his suffering as a stabbing, intermittent pain and sharp, shooting, and electric shock-like. Attacks were mainly provoked by chewing and talking. The patient experienced eight to ten attacks a day which lasted from a few seconds up to five minutes, generally remitting during the night, with pain free intervals that lasted from a few days up to fifteen days. Family history revealed relevant depressive syndrome in the father with repeated hospitalizations.

Since he revealed his psychological discomfort, which reflected on his private and professional life, sometimes affecting his concentration abilities while working, a psychiatric consultation was also scheduled.

The patient confided to the psychiatrist his uneasiness and reported that he was unsettled and tense: he experienced discomfort and anxiety due to worries about recurrence of pain. He also revealed his state of depression, especially originating from rumination about the condition.

As part of the current episode of care, after a combination of systemic medications had failed to ameliorate an assumption of maxillary sinusitis, the patient underwent an endodontic procedure, reportedly to reduce patient's suffering and complaints. A root canal therapy was carried out on his second maxillary right molar, the tooth considered by the patient as the cause of his pain. Since the pain had not alleviated during the four months following the procedure, the patient eventually decided to refer to our dental office for consultation.

*Patient assessment*

We visited the patient and we did not detect signs of gingival inflammation, or radiographic signs of other pathologies, on the bases of a orthopantogram and of a periapical X-Ray of the second upper right molar. The first upper right molar had undergone endodontic treatment. Percussion of quadrant one and four was negative. Intra-oral palpation did not elicit pain. In quadrant one, the second premolar was an implant that had been *in situ* for four years; the first premolar, canine and incisors all responded within normal limits when tested with cold for pulpal vitality. Occlusion was also checked and was well balanced with no pain during masticatory muscle palpation. Temporomandibular joints were pain-free during palpation or function and had a normal range of motion. A contrast-enhanced MRI was prescribed on July 2018 and the patient underwent the medical examination on September 2018. No signs of compression or alteration in trigeminal nerves were seen, suggesting an idiopathic form of TN. Pain intensity was investigated using the short-form McGill pain questionnaire (SF-MPQ). ^27^

It was immediately realized that we were dealing with an enigmatic pain, probably neuropathic in origin, and a complex and delicate situation, where quick and correct diagnosis seemed to be the principal goal.

*Diagnosis and therapeutic intervention.*

As pain was radiating distant from the tooth area to the ipsilateral region and following the distribution of the branches of the trigeminal nerve, a regimen of an increasing doses of carbamazepine (starting dose: 100 mg. twice a day for one week; then 200 mg three times a day for another week), up to 200 mg three times daily, was prescribed to the patient for two weeks. Unfortunately, this only led to slight pain reduction (SF-MPQ: score: from 3 to 2) and more psychological suffering and concerns of the patient about the condition. We then immediately discussed the psychological aspects of the patient and decided to prescribe a more tailored and patient-centered therapy: psychological, behavioural and psychopharmacologic approaches were modulated based on the patient’s psychological profile.

In addition, carbamazepine therapy was maintained, following the same regimen. We based our diagnostic method on the anamnesis, on the comprehensive physical examination, on the X-ray examination and on the specific psychiatric consultation. In addition to this and despite low efficacy, carbamazepine was also a useful diagnostic tool, because it was able to somewhat reduce pain intensity (SF-MPQ: score 2) and characteristics.

Based on the physical examination, the account of pain intensity and distinctive features of the pathology, the report of the state of anxiety and depression, the absence of any noticeable radiographic signs of pathology, and the slight improvement of pain intensity (SF-MPQ: score 2) and characteristics after carbamazepine had been prescribed, we concluded that we were dealing with a neuropathic pain and specifically with a case of TN.

TN is one of the most disabling orofacial pain conditions and the prognosis widely depends on the aetiology of the problem. ^31^

After a regimen of an increasing dose of carbamazepine up to 200 mg three times daily for two months had failed to completely quell the pain (SF-MPQ: score 2), psychological intervention was added (one session per week with a psychotherapist for three months, then reduced to one session every fifteen days up to now) and a regimen of three drops per day of citalopram, up to seven drops in the next ten days (starting dose: three drops per day for two days, then one more drop per day every two days) , and five drops of clonazepam in the evening for two months (then gradually reduced until suspension: reduced to three drops in the evening for one week and then suspended) were prescribed.

According to the diagnosis of TN, we recommended the patient to adhere to his carbamazepine prescription too (200 mg three times daily).

The rationale behind the decision to change our intervention was primarily based upon medical and psychological history; however, carbamazepine was maintained during treatment since it was deemed to be consistent in a clinical case of TN.

*Follow-up and outcomes*

The patient experienced relief of pain (SF-MPQ: score 1) and mood symptoms with a subjective perception of a satisfactory quality of life, which pleased the dental team.

After drug prescription, the patient was followed up every 15 days and was asked about pain intensity (SF-MPQ: score from 1 to 0) and characteristics and psychological suffering.

After two months, since he reported decisive improvements in symptoms (SF-MPQ: score 1-0) and psychological suffering, he was required to gradually reduce the therapy of antianxiety drugs until suspension.

Three months later, after he had confirmed decisive ameliorations in pain intensity (SF-MPQ: score 1-0) and features and psychological suffering, he was prescribed to reduce the dosage of antidepressant drug (citalopram drug) from seven to five drops per day. The same regimen of carbamazepine was maintained (200 mg three times daily).

The patient has reported mild symptomatology since then, but his uneasiness during pain attacks has manifested as fear of pain recurrence: due to these reasons the patient is now in a 15-day follow-up programme with the psychiatrist and is also currently under a regimen of five drops of citalopram per day and he is still adherent to a reduced dosage of 200 mg of carbamazepine twice a day. Subsequently, we also included the patient in a six month-recall programme to accomplish dental hygiene.

Phone calls, e-mails, and phone text messages, besides visits, were extremely efficient tools to assess patient prescription adherence and tolerability.

We report no adverse or unanticipated event in regard to the described clinical case.

When facing a patient with comorbid psychiatric disorders associated with a neuropathic pain, the dental team should preserve patient confidence, reduce anxiety and depression and obtain compliance. In addition to conventional therapies, the dentist should be prepared to supplement a behavioural approach, a psychiatric consultation and a pharmacologic treatment to adopt appropriate patient-centered, modulated and balanced medical care. Thus, a psychiatrist should be available as part of the dental team.

## Discussion

Orofacial pain always represents a demanding and stimulating situation for the practitioner. Treatment can be troublesome. ^2^ Long lasting pre-existing pre-treatment pain can represent a risk factor for the pain to become chronic. ^3^

Persistent pain tends to magnify the degree of pain and pain characteristics ^2^ and can cause stress in both the patient and the clinician.

Eventually, lack of knowledge, diagnostic procrastination and possible useless irreversible dental treatment can lead to frustration ^4^ up to real psychological distress, becoming a more complex condition to be managed.

Dealing with a wide range of patients, from the relaxed and collaborative to the anxious and depressed, the dental team should have a patient-centered approach and optimize and tailor the treatment, considering patients’ psychological profiles, pathology and pain characteristics. Therefore, a comprehensive anamnesis, including psychological assessment, and history listening is necessary and has been advocated. ^3^^,^^32^ Drug history also has to be included. ^32^ The proposed psychological, behavioural and psychopharmacologic approach has demonstrated advantages to control pain intensity and peculiarities in AO and TN case reports, drastically reducing pain duration over time, ameliorating the clinical scenario and improving the patient’s psychological profile with patient satisfaction.

The limitation in our approach to these cases is that the evaluation and treatment of the comorbid psychiatric disorders were made by scheduled psychiatric consultation, clinical observation and patient self-report, but were not based on a standardized evaluation scale or a questionnaire, since these are not habitually available in a dental office.

The diagnostic process might be particularly difficult, especially in cases of neuropathic pain with a non-odontogenic basis. ^1^^,^^2^

Moreover, the medical diagnosis can vary tremendously if a patient with pain below the imaginary line drawn between the eyes is assessed by a dentist or another medical specialist: in fact, orofacial pain in a dental environment is probably attributed to dental pathology, in contrast to orofacial pain patients in another medical environment likely being referred to a neurologist or to a maxillo-facial surgeon. ^32^

In regard to the diagnostic process, a comprehensive record of pain history and extraoral and intraoral examination of the head and neck region is mandatory. Laboratory investigations and imaging can sometimes be helpful. ^32^

Eventually, for an overview of the pathologies related to the challenging differential diagnosis in orofacial pain, several factors have to be taken into account.

As a general summary, comorbid psychiatric disorders are more frequent clinical findings in patients whose pathologies have shifted from an acute form to a chronic condition ^16^ and all types of diagnosed orofacial pain are more prevalent in females than males, ^2^ with myofascial pain remarkably more frequent. The peak age of prevalence is 50-67. Manual palpation of the muscles must be bilateral. The pain is generally acute and can be unilateral or bilateral. Careful investigation of potential myofascial trigger points seems to be of paramount importance in migraine-associated neck and shoulder muscle pain. ^33^ Mental health comorbidity has been recently investigated. ^34^

Myofascial pain usually positively responds to benzodiazepines and muscle relaxants ^2^ and this may help in the diagnostic process.

Temporomandibular disorders are also predominant in females ^35^ and are very frequent, affecting 5% to 12% of the population. The peak age of prevalence is 20-40. ^32^ Pain is related to the muscles used for mastication and of the neck. The pain is usually bilateral and all clinical investigations must be performed bilaterally. Clicking, locking, crepitus and limited opening (<40 mm) may be present and can lead to correct diagnosis. ^32^ Imaging of both joints can be helpful. Generally, with an acute onset, comorbid psychiatric disorders can increase the risk of chronicity. Hard full arch splints are advantageous. Non-steroidal anti-inflammatory drugs, benzodiazepines ^32^ and muscles relaxants have been considered controversial as medications. In a more recent study the above-mentioned medications and opioids, corticosteroids, anticonvulsants, anxiolytics and antidepressants were considered efficacious in alleviating pain. ^36^

Temporal tendinosis is an underestimated musculoskeletal pathology. ^37^ It is a chronic condition and causes orofacial pain.

There is still a lack of agreement on the most effective therapeutic management. Clinically, it can appear as unilateral facial pain accompanied, or not, by temporal headache; the second, most frequent clinical presentation is orofacial pain radiating from the distal temporalis tendon to the temporalis muscle.

Despite symptom similarity with temporomandibular disorders and giant cell arteritis, temporal tendinosis should be identified by means of anamnesis, proper related history, physical examination and dedicated imaging, such as ultrasound or MRI. ^37^

Dental causes evoke acute pain and they are in all probability unilateral. Dental caries and periodontal diseases affect approximately 20-50% of the world’s population. Both pathologies represent the main reason for tooth loss. ^38^

In most cases, the involved tooth is identifiable by the patient. Other times, e.g. in cases of pulpal involvement, the pain is more radiating and difficult to pinpoint to a specific tooth. 

A simple initial screening by periapical X-ray is very effective in the diagnostic process in the case of a decayed tooth, or to evaluate the alveolar bone and to recognize a periodontal disease. A comprehensive periodontal evaluation by gingival sulcular probing depth should always be undertaken.

For therapeutic or prophylactic reasons antibiotics are still largely prescribed to these patients, despite the known increasingly critical situation of antimicrobial resistance. ^38^

Non-steroidal anti-inflammatory drugs are also widely used for pain relief.

An accurate intraoral inspection to detect lesions related to diseases of the oral mucosa is also mandatory. ^39^ A histological examination of the oral mucosae can be supportive to more specifically identify a suspected pathology.

Maxillary sinusitis can be acute or chronic. Generally unilateral, it can also be bilateral. There is not age nor gender prevalence. There is an odontogenic and a non-odontogenic form. ^40^ The acute form is usually accompanied by pain and slight to moderate swelling of the cheek. Extraoral palpation of the skin, or intraoral palpation of the mucosa of the maxillary sinus area may provoke slight pain. Acute sinusitis is usually due to bacteria ^40^ and viruses. If a bacterial infection is suspected, it is advisable to prescribe antibiotics, decongestants, and nasal saline solution rinses. In cases of acute sinusitis related to pathologies affecting the premolars or molars, proper dental care therapy solves signs and symptoms. Acute sinusitis can also follow dental extractions. In these cases, possible oral antral fistula must be identified and all surgical efforts need to be done to close the fistula. ^32^

Chronic sinusitis is not usually associated with pain. Transnasal endoscopy done by an otolaryngologist is likely to be a useful and quick method for the diagnostic process.

In salivary gland disorders, a reduced volume of secretion or a change in the chemical composition of the saliva may be caused by salivary gland disfunction and can affect 5% to 46% of the population. ^41^ This is usually a chronic condition, difficult to treat, known as xerostomia and may negatively affect patients’ quality of life, thus causing psychological suffering. Acute pain in the region of the salivary glands can be elicited by salivary stones, characteristically at the sight of food or immediately before eating. In the case of tumours or duct obstruction of a salivary gland, pain in the trigeminal nerve can follow. Bimanual palpation can allow the clinician to recognize the stone. More frequently, it is a unilateral condition and can be non-invasively diagnosed by ultrasounds and possibly with associated sialendoscopy ^42^ or imaging.

Many new cases of AO following relatively common dental procedures have been reported in industrialized countries. It is an increasingly recognized condition affecting up to 6% of patients after they have undergone endodontic therapies ^13^ that has as an endpoint therapy the extraction of the involved tooth, without recovery of the pain. ^11^

It can be diagnosed in both sexes in adulthood, although women around mid-40s are singularly more affected by the condition. ^43^ It is a chronic condition, sleep can be undisturbed and pain can remit, with pain-free intervals, during the day. ^1^ It is a neuropathic pain, ^32^ characterized by continuous toothache following root canal therapy, apicoectomy, tooth extraction, implantology and even local analgesia administration. ^11^^,^^44^^–^^46^ AO can also follow facial trauma and inferior alveolar nerve block. ^47^ Poor analgesia at the time of the dental procedure has been regarded as an etiologic factor. ^32^

Prior long history of pain seems to have paramount importance since may increase the possibility of progression and origination of orofacial neuropathic pain in the same area. ^3^

Characteristically, patients with AO describe the pain as continuous, non-paroxysmal, throbbing, sometimes burning or stabbing, ^11^^,^^15^ which may make the differential diagnosis with trigeminal neuralgia difficult. The pain is referred to teeth or to the alveolar process, in the absence of any identifiable dental cause on clinical or radiographic examination. ^11^

Maxillary molars and premolars are more frequently affected. ^43^

Pain can spread and be diffused unilaterally or bilaterally ^44^ and thus difficult to localize for the patient. ^47^ Chronicity is at the base of demoralization, but it is unclear if this is the cause or the effect of the condition. ^44^

Amitriptylina has been reported to be helpful in treating AO, ^1^^,^^11^^,^^16^ but an association with cognitive-behavioural therapy is highly recommended. ^2^

TN is the most frequent cause of orofacial neuralgia, affecting four to five persons per 100,000 people with the highest prevalence in women, with a reported proportion in women and men of three to one, aged between 37 and 67. Pain follows the distribution of one or more branches of the trigeminal nerve with a predilection for the maxillary and mandibular branches. ^1^

It is a chronic condition. The pain is described as sudden, usually unilateral, brief, severe and stabbing. It can also be presented as a shooting, burning or paraesthesia sensation. ^12^

These paroxysmal attacks can last seconds to minutes. Other variants report pain for hours. Patients may experience as many as 10 to 30 attacks daily, although attacks may remit for weeks or months. ^32^ Pain rarely occurs during sleep. ^12^

Trigger points are characteristics of TN and attacks are provoked by light touch, washing, cold wind, eating, brushing teeth, talking, chewing. ^12^

As a result of the fact that carbamazepine is very often able to alleviate the pain, the logical conclusion in the diagnostic process of these clinical cases might be that the pathology faced and tackled by the practitioner might match the criteria of TN.

Although carbamazepine and oxcarbazepine remain the most commonly used drugs and are sometimes used as diagnostic tools, many others have therapeutic coherence. ^18^^,^^48^ As regards the second line drugs in classical or idiopathic TN, lamotrigine, baclofen, pimozide, tizanidine, tocainide, calcium channel blockers, levetiracetam, eslicarbazepine, local analgesics and sumatriptan have been studied and suggested. There is a lack of knowledge about drugs in secondary TN and in TN with concomitant continuous pain. ^48^ In cases of unbearable drug side-effects or uncontrolled pain, surgical management should be considered, ^1^ even if possibly followed by complications. TN has a profound impact on quality of life of affected patients. ^1^

For these reasons a multidisciplinary approach can be helpful for the management of the neuropsychologic aspect of chronic pain, such as in TN. ^49^

A branch of the trigeminal nerve can also be involved in a neuro cutaneous viral infection, known as herpes zoster, which can sometimes lead to trigeminal post-herpetic neuralgia. It is a fairly frequent event in the elderly and immunocompromised patients, and is less frequently observed amongst children. ^50^

The distribution of vesicles along nerves represents a diagnostic aid. Differential diagnosis can involve herpes simplex virus infection, recurrent aphthae, lichen planus, pemphigoid, pemphigus and immune defect consequent to drugs. ^50^

Although glossopharyngeal neuralgia may mimic TN due to paroxysmal pain attacks of two seconds to minutes, recurrent throughout the day, characteristically remitting for weeks or months, a difference in pain location is advantageous for the differential diagnosis: in fact, the pain is usually unilateral deep in the ear and/or back of the tongue, tonsils or neck. ^51^

It may be confused at the beginning with a temporomandibular disorder because pain is referred in the auditory meatus, ^32^ but a description of the pain as sharp, shooting electric shock, moderate to very severe, and the presence of evoking factors such as swallowing, coughing and touch of an ear, are likely to lead to the diagnosis of glossopharyngeal neuralgia.

Syncope is a rare complication due to anatomical propinquity with the vagus. ^32^

MRI may be indicated to identify areas of vascular compression and surgery may be used to treat the condition. ^52^

Percutaneous radiofrequency thermocoagulation is another option to treat glossopharyngeal neuralgia. ^53^

The trigeminal autonomic cephalalgias are a group of unilateral episodic pains ^32^ characterized by prominent headache and ipsilateral cranial signs controlled by the autonomic nervous system, like conjunctival injection, lacrimation, tearing and rhinorrhoea. Some trigeminal autonomic cephalalgias share their short-lasting painful characteristics with TN and thus they must be distinguished and eventually treated differently. ^54^

Affecting the oral cavity, burning mouth syndrome is a ubiquitous oral rare chronic condition with a burning sensation of the oral mucosa and tongue, without relation to clinical causes; a unique and elucidating symptom for a prompt diagnosis. ^55^ The syndrome usually affects peri- and post-menopausal women ^32^ often wearing removable prosthesis.

This condition is often chronic and, due to unexplained oral symptoms, the patient usually experiences psychological distress and frustration, as it happens in neuropathic pain patients. Reassurance of no worsening of the symptoms can act as a helpful factor.

Giant cell arteritis is the most frequent primary vasculitis of the elderly.

Patients complain about pain in the temporal region and this fact can be confusing, leading to a misdiagnosed temporomandibular disorder or vice versa.

If not rapidly treated, giant cell arteritis can result in blindness and sometimes in stroke with associated extreme pain of part or of the whole face.

Temporal artery biopsy as a diagnostic test is recommended, as well as other laboratory examinations. Steroids are the most credited therapy for patient management, but other efficacious therapies are now available. ^32^

Persistent idiopathic facial pain (PFIP), previously termed atypical facial pain, is a chronic condition and a rare disorder with an incidence rate of 4.4 per 100.000 people per year. ^56^ Females are more affected by the condition compared to males and the mean age of onset is in the mid 40s. ^57^

The International Classification of Headache Disorders, 3rd edition, published by the Headache Classification Committee of the International Headache Society (IHS) ^14^ presents PIFP as a continuous daily pain, lasting for more than two hours per day over a period of more than three months, but in the absence of clinical neurological deficit. Rarely, some patients report hours or days without pain. The pain is described as dull, aching, burning, throbbing and often stabbing and sharp. The pain is difficult to localize; most of the time it is radiating and unilateral, but sometimes bilateral. ^57^

Comorbid psychiatric disorders and psychosocial impairments have frequently been associated with PIFP. ^32^

Due to the complexity of the pathophysiology of the condition and comorbid psychiatric disorders, it has been concluded that an interdisciplinary approach is mandatory for the diagnostic process and management. ^58^ Treatment may include tricyclic antidepressants; more recent antidepressants such as duloxetine ^59^ and venlafaxine; ^60^ anticonvulsants; ^61^ low-level laser treatment; ^62^ and high-frequency repetitive transcranial magnetic stimulation. ^63^ From a psychological standpoint, it is important for the patient that the pain is acknowledged by the clinician as real. ^32^

Since comorbid psychiatric disorders may be frequently associated with orofacial pain, due to the effect of emotional states on pain perception and modulation, psychiatric and/or psychological counselling and proper drug management, together with an empathic attitude, might be determinant in patient compliance and an improvement in the clinical condition.

Moreover, the diagnosis and treatment of orofacial pain, especially if with a non-odontogenic basis, is difficult and challenging for the dental practitioner and, thus, they must be familiar with the signs and symptoms related to these conditions.

Dentists need to be well trained in this specific field to avoid diagnostic delays and multiple, irreversible and ineffective dental treatments.

It is imperative that dentists have a patient comprehensive, health-centered approach during the differential diagnostic process, refraining from focusing on ordinary, common sources of tooth pain, thus aggravating the clinical condition of the patient and exposing themselves to the risk of legal claims.

## Data availability

All data underlying the results are available as part of the article and no additional source data are required.

## Consent

Written informed consent for publication of their clinical details was obtained from the patients.
