# Atypical odontalgia and trigeminal neuralgia: psychological, behavioral and psychopharmacological approach in a dental clinic – an overview of pathologies related to the challenging differential diagnosis in orofacial pain

**DOI:** 10.12688/f1000research.51845.3

**Published:** 2022-06-27

**Authors:** Riccardo Tizzoni, Marta Tizzoni, Carlo Alfredo Clerici

**Affiliations:** 1 Independent Reasercher, 20122 Milano, Italy; 2 Department of Oncology and Haematology-Oncology,, University of Milano, 20122 Milano, Italy; 3 Clinical Psychology Unit, Fondazione IRCCS, Istituto Nazionale dei Tumori, 20133 Milano, Italy

**Keywords:** atypical odontalgia, trigeminal neuralgia, case report, psychiatric disorders, orofacial pain

## Abstract

Orofacial pain represents a challenge for dentists, especially if it does not have an odontogenic origin. Orofacial neuropathic pain may be chronic, is arduous to localize and may develop without obvious pathology. Comorbid psychiatric disorders, such as anxiety and depression, coexist and negatively affect this condition. This article presents one case of atypical odontalgia and one of trigeminal neuralgia treated with psychological and psychopharmacological tailored and adapted therapies, after conventional medications had failed. Additionally, an overview of the pathologies related to the challenging differential diagnosis in orofacial pain is given.

A 68-year-old man complained of chronic throbbing and burning pain in a maxillary tooth, which worsened upon digital pressure. Symptoms did not abate after amitriptyline therapy; psychological intervention along with antianxiety drug were supplemented and antidepressant agent dosage were incremented. The patient reported improvement and satisfaction with the multidisciplinary approach to his pathology.

A 72-year-old man complained of chronic stabbing, intermittent, sharp, shooting and electric shock-like pain in an upper tooth, radiating and following the distribution of the trigeminal nerve. Pain did not recur after psychological intervention and a prescription of antidepressant and antianxiety agents, while carbamazepine therapy had not been sufficient to control pain. Due to concerns with comorbid psychiatric disorders, we adopted a patient-centered, tailored and balanced therapy, favorably changing clinical outcomes.

Comorbid psychiatric disorders have a negative impact on orofacial pain, and dentists should consider adopting tailored therapies, such as psychological counselling and behavioral and psychopharmacologic strategies, besides conventional treatments. They also must be familiar with the signs and symptoms of orofacial pain, obtaining a comprehensive view of the pathologies concerning the differential diagnosis. A prompt diagnosis may prevent pain chronicity, avoiding an increase in complexity and a shift to orofacial neuropathic pain and legal claims.

## Introduction

The diagnosis and treatment of pain represent two of the most challenging tasks in clinical dental practice, especially if pain is not odontogenic. ^1^ Particular attention should be paid to orofacial disorders, which must be treated thoroughly due to their broad aesthetic, biological, emotional and social implications for the patient. ^2^ Overall, the prevalence of orofacial pain is significantly higher in females than in males. ^2^ A long-lasting and untreated inflammatory pain may evolve into an orofacial neuropathy. ^3^ Therefore, it is important to reach an effective identification of the underlying pathology and promptly counteract it. Timely intervention will prevent the shift to a chronic pain condition, which may lead to psychological distress. ^2^ A final psychological comorbidity can lead the patient to experience enhanced pain intensity. Generally, comorbid psychological disorders are more frequent in patients whose symptoms have shifted from acute to chronic. ^4^

Successful treatment of orofacial pathologies depends on an accurate differential diagnosis. This is the process of logically analyzing the patients’ symptoms and medical history, along with the results of physical, laboratory and other tests, based on the clinician’s medical knowledge. Differential diagnosis allows discriminating among different diseases which partially share signs or symptoms, and it can mediate an effective treatment of the pathology causing orofacial pain. This prevents pain chronicization, thus avoiding diagnostic delays and unnecessary and purposeless dental treatments ^5^ which may also increase the risk of legal litigations. Nonetheless, differential diagnosis is often difficult for dental practitioners, as radiographic signs and clinical symptoms of many pain-causing oral pathologies ^6^ ^,^ ^7^ are often absent. In turn, they should be very well trained and aware of the difficulties of the differential diagnostic process.

Late diagnosis, irreversible dental treatments and a lack of knowledge may lead the patient to lose confidence and hope, resulting in frustration that may lead to clinical depression. ^5^ ^,^ ^8^ The prevention, early diagnosis and treatment of inflammatory pain may thus help avoid the development of neuropathic pain, ^3^ consequent anxiety ^2^ ^,^ ^9^ and even more severe psychological impairments. ^5^ ^,^ ^8^ Anxiety is a mental state that arises spontaneously and unconsciously and is characterized by stress, worried thoughts and physical changes. ^10^ Depression, instead, is defined as a “ *medical condition that includes abnormalities of affect and mood, neurovegetative functions (such as appetite and sleep disturbances), cognition (such as inappropriate guilt and feelings of worthlessness), and psychomotor activity (such as agitation or retardation)*”. ^11^

Considering the strict relationship between neuropathic orofacial pain and the possible onset of psychological disorders, dental practitioners should be well trained not only to correctly identify this pathology but also to proactively identify and measure anxiety and/or depression. A correct assessment of anxiety intensity may allow preventing the occurrence of more severe psychological pathologies. However, anxiety is not easy to measure. Dentists may rely on different methods, such as using the modified dental anxiety scale (MDAS). ^12^ To assess depression, the Beck depression inventory - version two (BDI-II) is as a cost-effective and reliable tool with broad applicability both for clinical research and practice, allowing an easy discrimination between depressed and non-depressed subjects. ^13^

When a patient presents with a non-odontogenic orofacial pain, and associated psychological comorbidities are suspected, a thorough psychological assessment is mandatory. After an anxiety or depression diagnosis, the patient may be first prescribed appropriate drugs for both the pain and any psychological co-morbidities. As a rule, it is important to reassure patients by always providing an explanation of their pain and comfort them with professional empathy. However, if the pain does not subside, a more patient-centered and comprehensive approach may be required. In such cases, well-tolerated effective antidepressant and antianxiety treatments along with counselling and psychological therapies should be considered.

### Aims of the study

Despite available data on single forms of orofacial pain, different pain types arising from regions of the face and mouth remain understudied. Such information would enable the familiarization of dental practitioners with the signs and symptoms of orofacial pain, especially those with non-odontogenic origins. ^14^ Therefore, our first aim was to provide an overview of the most common orofacial pathologies which dental practitioners may encounter during their activity. This overview is not meant to be a comprehensive and fully exhaustive description of each pathology. Rather, the information we provide may offer a synthetic guide to support the differential diagnostic process, which is often complex, especially when assessing non-odontogenic pain. In our description, we specifically focus on two conditions, atypical odontalgia (AO) and trigeminal neuralgia (TN), which are the most diagnosed neuropathic pains in dental practice.

We discuss that a prompt and correct assessment of the pathology can help prevent the development of chronic conditions and, in turn, psychological disorders. Supporting this, we describe two case reports dealing with AO and TN. Our cases well demonstrate that late diagnosis can lead to comorbidities. In these occasions, psychological treatment may be key for the successful healing of chronic neuropathies. In line with our discussion, we stress the importance of a new patient-health-centered approach, which is required during a differential diagnosis to properly identify pathologies, thus preventing the development of chronic pains and possible comorbidities due to psychological disorders.

### Orofacial pathologies in dental practice – An overview

Atypical odontalgia (AO), also known as persistent dentoalveolar pain disorder, ^5^ is a form of chronic dental pain not associated with any other pathological sign. ^15^ AO is one of the most frequently diagnosed neuropathic pains ^[^ ^1^ ^]^ ^2^ ^,^ ^15^ ^,^ ^16^ together with trigeminal neuralgia (TN). It affects adults of both sexes, although females around their mid-40s seem to be more prone to develop it. ^17^

AO can affect up to 6% of patients subjected to endodontic therapies. ^5^ ^,^ ^18^ Usually, tooth extraction does not allow a recovery from pain. ^6^ Maxillary molars and premolars are more frequently involved. ^17^ Typically, patients with AO describe their pain as continuous, non-paroxysmal, throbbing and sometimes burning or stabbing. ^6^ ^,^ ^15^ This makes AO difficult to distinguish from TN in the differential diagnosis. Pain can spread unilaterally or bilaterally, ^19^ thus being difficult to localize for the patient. ^20^ Alternatively, it can refer to the teeth or the alveolar process, in both cases with no identifiable dental causes on clinical or radiographic examination. ^6^ Paresthesia or dysesthesia may be detected during examination, ^1^ and sometimes touching the area may worsen the pain. If this orofacial pain is not promptly treated, the progression of its severity in the same area is more likely. ^3^

The pathophysiological mechanisms underlying the onset and persistence of this condition have not been fully elucidated. ^5^ The current hypothesis is that AO is neuropathic, as injuries to teeth and/or periodontal tissues may alter the periodontal nerve plexus, resulting in peripheral sensitization. ^15^ AO can indeed arise as a continuous toothache after root canal therapy, apicoectomy, tooth extraction, implantology and even local analgesia administration. ^6^ ^,^ ^19^ ^,^ ^21^ ^,^ ^22^ Additionally, it can follow facial trauma and inferior alveolar nerve block. ^20^ Poor analgesia at the time of the dental procedure has been regarded as an etiologic factor. ^16^

Chronic AO is often concurrent with demoralization, but whether this is the cause or the effect of the condition is still debated. ^19^ In turn, coexisting psychological disorders should be also taken into account. In a recent study, ^4^ 46.2% of patients with AO showed comorbid psychological disorders. Among them, 15.4% showed depression and 10.1% were anxious, while serious mental disorders like bipolarity and schizophrenia were present only in 3.0% and 1.8% of the cases, respectively. Therefore, pain may have a significant emotional basis in addition to the sensory ones we previously discussed. ^4^ For these reasons, amitriptyline has been reported to be helpful in treating AO, ^1^ ^,^ ^4^ ^,^ ^6^ but its combination with cognitive-behavioral therapy is highly recommended. ^2^

Table 1 reports the diagnostic criteria for the diagnosis of both AO ^23^ and persistent idiopathic facial pain (PIFP), previously defined as atypical facial pain. ^24^

**Table 1.** Diagnostic criteria for the diagnosis of Atypical Odontalgia and Persistent Idiopathic Facial Pain according to the International Classification of Headache Disorders (ICHD-3 classification ^23^).

Atypical Odontalgia		The term has been applied to a continuous pain in one or more teeth or in a tooth socket after extraction, in the absence of any usual dental cause.
		This is thought to be a subtype of Persistent idiopathic facial pain although it is more localized, the mean age at onset is younger and genders are more balanced.
		Based on the history of trauma, atypical odontalgia may also be a sub-form of Painful post-traumatic trigeminal neuropathy.
		These subtypes/forms, if they exist, have not sufficiently studied to propose diagnostic criteria.
Persistent idiopathic facial pain (PIFP)	A	Facial and/or oral pain fulfilling criteria B and C
	B	Recurring daily for >2 hours/day for >3 months
	C	Pain has both of the following characteristics: 1. poorly localized, and not following the distribution of a peripheral nerve; 2. dull, aching or nagging quality
	D	Clinical neurological examination is normal
	E	A dental cause has been excluded by appropriate investigations
	F	Not better accounted for by another ICHD-3 diagnosis

Trigeminal neuralgia (TN) is the most frequent (82.1%) cause of orofacial neuralgia among patients with neuropathic pain, ^2^ affecting four to five individuals per 100,000. The highest prevalence is observed in females, with a reported proportion of women to men of three to one, in people aged between 37 and 67.

TN is a chronic condition. Pain follows, usually unilaterally, the distribution of one or more branches of the trigeminal nerve, with a predilection for the maxillary and mandibular branches. ^1^ Its symptoms are similar to odontogenic ones, ^1^ with paroxysmal, sudden, sharp, brief, severe and stabbing pain, sometimes associated with shooting, burning or paresthesia sensations. ^7^ The paroxysmal attacks can last seconds to minutes or even hours. Patients may experience 10 to 30 attacks daily, although pain may remit for weeks or months, ^16^ ^,^ ^25^ rarely occurring during sleep. ^7^ Trigger points are characteristically localized around the nose and the mouth. Typically, in TN, pain attacks are provoked by light touch, washing, cold wind, eating, brushing teeth, talking and chewing. ^7^

TN affects 1% of patients with multiple sclerosis, and 2–8% of patients with TN are affected by multiple sclerosis. ^26^ The disruptive features of TN pain often have unfavorable effects on daily life, with a consequent poor quality of life and significantly reduced working performance. ^27^ For these reasons, as for AO, the psychological aspects of patients affected by TN should also be carefully assessed. Its pathophysiological mechanism is supposed to be a tumor or vascular compression that may lead to partial and focal nerve demyelination and consequent abnormal transmission and processing of impulses along the trigeminal nerve. ^1^ ^,^ ^26^ ^,^ ^28^ Paroxysmal pain is triggered by vascular compression (this is the usual cause of demyelination at the site just before the nerve enters the pons) and multiple sclerosis (this causes demyelination at the site just after entry into the pons). ^29^ A clinical and neuroimaging study reported the association of concomitant continuous pain with trigeminal nerve atrophy in up to 50% of patients with TN. In these cases, this type of pain is likely related to axonal loss and abnormal activity in denervated trigeminal second-order neurons. ^30^ Genetic factors and chronic irritation or trauma may also play a role in the pathophysiology of TN. ^1^ ^,^ ^29^ ^,^ ^31^

Recent diagnostic criteria classify TN as “classical” (related to neurovascular compression producing morphological changes on the trigeminal root), “secondary” (related to a major neurological disease) or “idiopathic” (with unknown etiology). ^29^ ^,^ ^31^ During diagnostic work-up, magnetic resonance imaging (MRI) may reveal superior cerebellar artery aneurysms ^32^ and venous compression, ^33^ but also benign or malignant lesions and plaques of multiple sclerosis. ^32^ Additionally, carbamazepine can be used as a diagnostic tool, because it can very often alleviate the characteristic pain associated with TN.

In addition to carbamazepine and oxcarbazepine, TN pain can be treated with several drugs. ^26^ ^,^ ^34^ In classical or idiopathic TN, lamotrigine, baclofen, pimozide, tizanidine, tocainide, calcium channel blockers, levetiracetam, eslicarbazepine, local analgesics and sumatriptan have been studied and suggested as second-line drugs. At present, there are no recommended drugs for the treatment of secondary TN or TN with concomitant continuous pain. ^34^ In cases of unbearable drug side-effects or uncontrolled pain, surgical management should be considered, ^1^ even if possibly followed by complications.

Table 2 reports the diagnostic criteria for the diagnosis of TN. ^23^

**Table 2.** **Diagnostic criteria for the diagnosis of Trigeminal Neuralgia (ICHD-3 classification** ^23^ **).**

Trigeminal neuralgia	A	Recurrent paroxysms of unilateral facial pain in the distribution(s) of one or more divisions of the trigeminal nerve, with no radiation beyond, and fulfilling criteria B and C
	B	Pain has all of the following characteristics: 1) Lasting from fraction of a second to two minutes 2) Severe intensity 3) Electric shock-like, shooting, stabbing or sharp in quality
	C	Precipitated by innocuous stimuli within the affected trigeminal distribution
	D	Not better accounted for by another ICHD-3 diagnosis
		Notes: 1) In a few patients, pain may radiate to another division, but it remains within the trigeminal dermatomes. 2) Duration can change over time, with paroxysms becoming more prolonged. A minority of patients will report attacks predominantly lasting for >2 minutes. 3) Pain may become more severe over time. 4) Some attacks may be, or appear to be, spontaneous, but there must be a history or finding of pain provoked by innocuous stimuli to meet this criterion. Ideally, the examining clinician should attempt to confirm the history by replicating the triggering phenomenon. However, this may not always be possible because of the patient’s refusal, awkward anatomical location of the trigger and/or other factors.

Herpes zoster is a viral infection that can sometimes lead to trigeminal post-herpetic neuralgia. It is frequent in the older people and immunocompromised patients, with children being less affected. ^35^ The distribution of vesicles along nerves represents a diagnostic aid. Differential diagnosis can involve herpes simplex virus infection, recurrent aphthae, lichen planus, pemphigoid, pemphigus and immune defects arising from drug use. ^35^

In addition to herpes zoster, myofascial pain is highly frequent. The peak age of its prevalence is 50–67 years. Manual palpation of the muscles must be bilateral for its diagnosis. The pain is generally acute and can be unilateral or bilateral. Careful investigation of potential myofascial trigger points seems to be of key importance in migraine-associated neck and shoulder muscle pain. ^36^ Mental health comorbidities have been recently investigated ^37^ in association to this pathology. Additionally, myofascial pain usually responds positively to benzodiazepines and muscle relaxants, ^2^ which may help in the diagnostic process.

Temporomandibular disorders are another frequent type of orofacial pain, affecting 5% to 12% of the population and predominantly occurring in females. ^38^ The peak age of their prevalence is 20–40 years. ^16^ Their characteristic pain is related to neck muscles and muscles used for mastication. It is usually bilateral and all clinical investigations must be performed bilaterally. Clicking, locking, crepitus and limited opening (<40 mm) may be present and can lead to a correct diagnosis1, ^6^ which can be helped by the imaging of both joints. Generally, with an acute onset, comorbid psychological disorders can increase the risk of chronicity. Hard full-arch splints are advantageous for this pain, while non-steroidal anti-inflammatory drugs, benzodiazepines ^16^ and muscle relaxants have been usually considered controversial as medications. However, in a more recent study, the above-mentioned medications and opioids, corticosteroids, anticonvulsants, anxiolytics and antidepressants were efficacious in alleviating this pain. ^39^

Temporal tendinosis is an underestimated musculoskeletal pathology. ^40^ It is a chronic condition that causes orofacial pain, and its most effective therapeutic management remains controversial. Clinically, it can appear as a unilateral facial pain possibly associated to temporal headache or as an orofacial pain radiating from the distal temporalis tendon to the temporalis muscle. Despite symptom similarity with temporomandibular disorders and giant cell arteritis, temporal tendinosis should be identified by means of anamnesis, proper related history, physical examination and dedicated imaging, such as ultrasound or MRI. ^40^

Among orofacial disorders, dental causes evoke acute pain and are most likely unilateral. Dental caries and periodontal diseases affect approximately 20–50% of the world’s population and represent the main reason for tooth loss. ^41^ In most cases, the involved tooth is identifiable by the patient. Otherwise, the pain is more radiating and difficult to pinpoint to a specific tooth, e.g., in cases of pulpal involvement. A simple initial screening by periapical X-ray is very effective in the diagnostic process in the case of a decayed tooth or to evaluate the alveolar bone and recognize a periodontal disease. A comprehensive periodontal evaluation by gingival sulcular probing depth should always be undertaken. For therapeutic or prophylactic reasons, antibiotics are still largely prescribed to these patients, despite the criticality of antimicrobial resistance. ^41^ Non-steroidal anti-inflammatory drugs are also widely used for pain relief. Additionally, an accurate intraoral inspection to detect lesions related to diseases of the oral mucosa is mandatory. ^42^ A histological examination of the oral mucosae can be supportive to identify a suspected pathology more specifically.

Maxillary sinusitis may be an acute or chronic pathology. It is generally unilateral, although it can also be bilateral. Its prevalence shows no age or sex differences. There is an odontogenic and a non-odontogenic form. ^43^ The acute form is usually associated to pain and slight to moderate swelling of the cheek. Extraoral palpation of the skin or intraoral palpation of the mucosa of the maxillary sinus area may provoke a slight pain. Acute sinusitis is usually due to bacteria ^43^ and viruses. If a bacterial infection is suspected, it is advisable to prescribe antibiotics, decongestants, and nasal saline solution rinses. In cases of acute sinusitis related to pathologies affecting the premolars or molars, proper dental care therapy can solve the signs and symptoms. Acute sinusitis can also follow dental extractions. In these cases, possible oral antral fistula must be identified, and all surgical efforts should be carried out to close the fistula. ^16^ Differently from the acute form, chronic sinusitis is a pathology that is not usually associated with pain. Trans-nasal endoscopy performed by an otolaryngologist is likely to be a useful and quick method for the diagnostic process.

Salivary gland disorders encompass a reduced volume of secretion or a change in the chemical composition of the saliva, as caused by salivary gland disfunction. They can affect from 5% to 46% of the population. ^44^ These conditions are usually chronic, which makes them difficult to treat. In turn, the life quality of their patients may be negatively affected, thus causing psychological suffering. Acute pain in the region of the salivary glands can be elicited by salivary stones, characteristically at the sight of food or immediately before eating. In the case of tumors or duct obstruction of a salivary gland, pain in the trigeminal nerve can follow. Bimanual palpation can allow the clinician to recognize the stone. More frequently, a unilateral condition is present and can be non-invasively diagnosed by ultrasounds and possibly with associated sialoendoscopy ^45^ or imaging.

Another possible orofacial pain pathology is glossopharyngeal neuralgia. It may mimic TN, because paroxysmal pain attacks of two seconds to minutes occur throughout the day, characteristically remitting for weeks or months. However, its pain is usually unilateral and characteristically deep in the ear and/or back of the tongue, tonsils or neck. ^46^ It may be confused at the beginning with a temporomandibular disorder because its pain is located in the auditory meatus. ^16^ However, a description of the pain as a moderate to very severe, sharp and shooting electric shock and the presence of evoking factors such as swallowing, coughing and the touching of an ear are distinctive for diagnosing glossopharyngeal neuralgia. Syncope is a rare complication due to anatomical propinquity with the vagus, ^16^ and MRI may be indicated to identify areas of vascular compression. Treatment involves surgery ^47^ or percutaneous radiofrequency thermocoagulation. ^48^

Trigeminal autonomic cephalalgias are a group of unilateral episodic pains ^16^ characterized by prominent headache and ipsilateral cranial signs controlled by the autonomic nervous system, like conjunctival injection, lacrimation, tearing and rhinorrhea. Some trigeminal autonomic cephalalgias share short-lasting painful characteristics with TN and thus must be distinguished and eventually treated differently. ^49^

Burning mouth syndrome is a rare and chronic condition affecting entire oral cavity. It mediates a burning sensation of the oral mucosa and tongue, without relation to clinical causes, which is a unique symptom for a prompt diagnosis. ^50^ The syndrome usually affects peri- and post-menopausal women ^16^ often wearing removable prosthesis. Due to unexplained oral symptoms, patients usually experience psychological distress and frustration, as occurs in patients with neuropathic pain. Reassurance of a lack of worsening of current symptoms can act as a helpful factor.

Giant cell arteritis is the most frequent primary vasculitis in the elderly. Patients complain about pain in the temporal region, which can be confusing and lead to a misdiagnosed temporomandibular disorder. If not rapidly treated, giant cell arteritis can result in blindness and sometimes in stroke, with associated extreme pain expanding to a part of or the whole face. Temporal artery biopsy and other laboratory examinations are recommended as diagnostic tests. Steroids are the most credited therapy for patient management, but other efficacious treatments are now available. ^16^

Finally, persistent idiopathic facial pain (PIFP)—previously termed atypical facial pain—is a chronic condition and rare disorder with an incidence rate of 4.4 per 100.000 people per year. ^24^ Women are more affected than men and the mean age of onset is mid 40s. ^51^ The International Classification of Headache Disorders, 3 ^rd^ edition, published by the Headache Classification Committee of the International Headache Society (IHS) ^23^ describes PIFP as a continuous daily pain, lasting for more than two hours per day over a period of more than three months, but in the absence of clinical neurological deficits. Rarely, some patients reported hours or days without pain. The pain is described as dull, aching, burning, throbbing and often stabbing and sharp. It is difficult to localize, being mostly radiating and unilateral, but sometimes is bilateral. ^51^ Comorbid psychological disorders and psychosocial impairments have frequently been associated with PIFP, ^16^ and their treatment may include tricyclic antidepressants and more recent antidepressants drugs, such as duloxetine ^52^ and venlafaxine, ^53^ or anticonvulsants. ^54^ Additionally, low-level laser treatment ^55^ and high-frequency repetitive transcranial magnetic stimulation ^56^ can be considered.

In the following section, two case studies are described to demonstrate: a) the importance of a promptly effective differential diagnosis and b) the importance of considering psychological treatments as part of the therapy. Our reports may be helpful for dental practitioners to broaden their clinical view and consider more information during their practice. A prompt diagnosis can prevent pain chronicity, avoiding an increase in complexity, a shift to orofacial neuropathic pain, the possible development of psychological comorbidities and even legal claims.

## Case presentation

### Atypical odontalgia

*Clinical presentation and history*

A Caucasian 68-year-old Italian man complaining about moderate pain in the second upper left premolar was referred to our private practice. He suffered from a perpetuated period of throbbing or burning pain in the tooth or in the alveolar process, also characterized by a tingling sensation upon digital pressure, with a troublesome feeling on his prosthetic zirconia crown. The tooth had undergone uneventful root canal therapy many years before, a big cast post had been inserted, and a gold alloy crown manufactured. The pain had been persisting for about six months, since a dentist had insisted on removing the old gold alloy crown from the tooth to make a new aesthetic zirconia crown. The pain was described as ongoing, but was absent during sleep, with pain-free intervals during the day. It had not been susceptible to non-steroidal anti-inflammatory drugs for six months.

His clinical history did not present relevant findings, and he did not report familial pathologies. At anamnestic psychiatric consultation, the patient reported a marked lack of concentration during working hours and considerable impact on his personal and social life. Additionally, the patient displayed anxiety and irritability, especially relating to the difficulties of the diagnostic process. Significant symptoms of depression were described by the patient as a consequence of his condition.

*Patient assessment*

A comprehensive analysis of the mucosae and gingivae was carried out in quadrants two and three: adjacent teeth showed normal responses at testing for vitality with cold. Normal and balanced occlusal points were found. The contact points of the crowns in the area showed no food impaction coexistence. Percussion of the teeth or intra-oral palpation of the above-mentioned quadrant did not provoke pain, except for the second maxillary left premolar.

The function of the temporomandibular joints was within the normal range of motion and no joint pain was present. A periapical X-ray of the second maxillary left premolar and neighboring teeth was also obtained. It showed a normal tooth and surrounding bone structure, with no signs of pathology. The neuropathic origin of the pain was excluded through neurophysiological tests (trigeminal reflexes) evaluating trigeminal afferent integrity. The diagnostic method was based on the anamnesis, comprehensive physical examination, X-ray examination and specific anamnestic psychiatric consultation. Pain intensity was investigated using the short-form McGill pain questionnaire (SF_MPQ). ^57^

*Diagnosis and therapeutic intervention*

Because the patient had been experiencing symptoms for several months, he insisted on having an appointment for tooth extraction, despite our clinical advice. He was then proposed to be rehabilitated by a single crown supported by an implant to be immediately loaded. After he signed a specific informed consent form for tooth extraction, and even though painful micro-fractures of the root were thought to be possibly due to the big post inserted within the root canal, we reluctantly extracted the tooth, paying attention not to damage the alveolar bone during the extraction. The implant seat was prepared according to the manufacturer’s instructions, and the implant (Biomet 3i, 15 × 4 mm) was placed, its primary implant stability being 25 Ncm. A temporary resin crown was then manufactured ^58^ and delivered to the patient. After six months, scanner-acquired 3D models allowed us to mill a CAD/CAM metal coping to be later veneered. Two months later, a cemented metal-ceramic crown was finally delivered to the patient. ^59^ After tooth extraction and implant placement, the patient was followed-up weekly for four weeks, and the pain did not remit. Although useless, tooth extraction was discriminating, as it allowed ascertaining that the tooth was not the cause of the pain. Additionally, due to the absence of any noticeable odontogenic etiology, and based on the psychological suffering reported by the patient and clinical and radiographical findings, the pain was deemed to be enigmatic in origin, thus leading to an AO diagnosis.

The prognostic characteristic of this pathology is generally thought to be a treatment-resistant condition, ^60^ but a multi-disciplinary approach to treatment can lead to positive outcomes. Because pain (SF-MPQ: score 2) had not remitted after a six-month non-steroidal anti-inflammatory drug period, nor after tooth extraction, we settled for a more patient-health-centered approach. We prescribed a combinatorial therapy consisting of psychological counselling with behavioral and pharmacological intervention. According to the literature and previous hypothesis of neurogenic pain (specifically in AO), the tricyclic antidepressant amitriptyline was prescribed. Nevertheless, symptoms did not abate (SF-MPQ: score 2) after three weeks of increasing the doses of amitriptyline (starting dose: 25 mg in the evening for one week; 25 mg in the morning and in the evening during the second week; 25 mg in the morning and 50 mg in the evening during the third week) up to 75 mg per day. Therefore, psychological intervention was added (i.e., psychological counselling and cognitive behavioral therapies, based on one session per week with a psychotherapist). Additionally, an increment of 25 mg of amitriptyline per week, up to 150 mg per day for six months (then gradually reducing 50 mg per week, until suspension was achieved within three weeks) was prescribed and five drops of clonazepam in the evening for one month (then gradually reducing to three drops for one week and then suspending it) were added.

The rationale for the changes in our professional intervention was based on the medical and psychological history, from which we assumed we were facing a case of AO associated with comorbid psychiatric disorders.

*Follow-up and outcomes*

After implementing psychological and behavioral supporting interventions, the patient reported satisfaction and improvement at each follow-up visit. Fortunately, dental extraction was not a precipitating event, and the clinical case was resolved, from a prosthetic standpoint, with the aid of implant-supported rehabilitation. The rehabilitation phases lasted eight months, from first-stage implant insertion surgery to the delivery of the implant-supported ceramic crown. The patient was followed up every 15 days by the dentist and the psychiatrist, and was asked about pain intensity (SF-MPQ: score from 1 to 0) and characteristics and psychological conditions. After six months, because he reported decisive improvements in symptoms (SF-MPQ: score 0) and psychological suffering, we prescribed him a gradually reduced regimen of antidepressant therapy until suspension. The patient has been pain-free since then, and is now in a two-month follow-up program with the psychiatrist and in a six-month recall program for dental hygiene.

Psychiatrist and dentist visits were interspersed with phone calls, e-mails or phone text messages to accomplish a comprehensive check of pharmacotherapy adherence and tolerability. Consequently, pain, symptoms and psychological conditions were also assessed. No adverse or unanticipated events were observed.

The diagnosis and treatment of this clinical case was challenging and difficult, even though gratifying for the successful differential diagnostic process, especially because the pain had a non-odontogenic basis, and due to the successful treatment that followed.

### Trigeminal neuralgia

*Clinical presentation and history*

A Caucasian 72-year-old Italian man suffered from a six-month history of pain of variable intensity, from moderate to severe, in the molar region of the right maxillary quadrant, radiating distant from the tooth area to the ipsilateral region and following the distribution of the branches of the trigeminal nerve. The patient had undergone a combination of systemic medications that had failed to ameliorate a putative maxillary sinusitis. After that, he had also been subjected to an endodontic procedure, reportedly to reduce his suffering and complaints. A root canal therapy had been carried out on his second maxillary right molar, the tooth considered by the patient as the cause of his pain. Because the pain had not alleviated during the four months following the procedure, he eventually decided to refer to our dental office for consultation.

The patient reported a variety of symptoms, and pain seemed to be odontogenic, as he insisted it originated from a single tooth, before radiating to the ipsilateral region. The patient described his suffering as a stabbing, intermittent, sharp, shooting and electric-shock–like pain. Attacks were mainly provoked by chewing and talking. He experienced eight to ten attacks a day which lasted from a few seconds up to five minutes, generally remitting during the night, with pain-free intervals that lasted from a few days up to fifteen days. Family history revealed that his father had had a relevant depressive syndrome with repeated hospitalizations. Because he revealed a psychological discomfort affecting his private and professional life (sometimes his concentration abilities while working were reduced), a psychiatric consultation was also scheduled. The patient confided his uneasiness to the psychiatrist and reported that he was unsettled and tense: he experienced discomfort and anxiety due to worries about pain recurrence. He also revealed his state of depression, especially originating from rumination about the condition.

*Patient assessment*

No signs of gingival inflammation were detected. A panoramic and a periapical X-Ray of the second upper right molar showed no radiographic signs of other pathologies. The first upper right molar had undergone endodontic treatment. Percussion of quadrant one and four was negative. Intra-oral palpation did not elicit pain. In quadrant one, the second premolar was an implant that had been *in situ* for four years. The first premolar, canine and incisors all responded within normal limits when tested with cold for pulpal vitality. Occlusion was also checked and was well balanced with no pain during masticatory muscle palpation. Temporomandibular joints were pain-free at palpation or on function and had a normal range of motion. The patient underwent a contrast-enhanced MRI showing no signs of compression or alteration in trigeminal nerves, suggesting an idiopathic form of TN. ^31^ Therefore, pain was diagnosed as enigmatic, while probably of neuropathic origin. The overall situation was deemed complex and delicate, calling for a quick and correct diagnosis. As for AO, the diagnostic method was based on the anamnesis, comprehensive physical examination, X-ray examination, and specific anamnestic psychiatric consultation. Pain intensity was investigated using the short-form McGill pain questionnaire (SF-MPQ). ^57^

*Diagnosis and therapeutic intervention*

Because pain was radiating distant from the tooth area to the ipsilateral region and following the distribution of the branches of the trigeminal nerve, a regimen of an increasing doses of carbamazepine (starting dose: 100 mg twice a day for one week; then 200 mg three times a day for another week), up to 200 mg three times daily, was prescribed for two weeks. ^61^ Unfortunately, this only led to a slight pain reduction (SF-MPQ: score: from 3 to 2) and enhanced psychological suffering and patient concerns about the condition. This led us to prescribe a more tailored and patient-health-centered therapy, adjusting psychological, behavioral and psychopharmacological approaches to the patient’s psychological profile. Cognitive behavioral therapy has been demonstrated to be an effective approach to improve life quality of TN patients. ^62^ Additionally, carbamazepine therapy was maintained, following the same regimen. Despite its low efficacy, carbamazepine was also a useful diagnostic tool, because it was able to somewhat reduce pain intensity (SF-MPQ: score 2) and characteristics.

Considering the physical examination, reported pain intensity, distinctive features of the pathology, reported state of anxiety and depression, absence of any noticeable radiographic signs of pathology and slight reduction in pain intensity (SF-MPQ: score 2) and characteristics after carbamazepine had been prescribed, we concluded that we were dealing with neuropathic pain, and specifically with a case of TN, one of the most disabling orofacial pain conditions whose prognosis widely depends on the etiology of the problem. ^63^ Increasing the dose of carbamazepine up to 200 mg three times daily for two months failed to completely suppress the pain (SF-MPQ: score 2). Therefore, psychological intervention was added (one session per week with a psychotherapist for three months, then reduced to one session every fifteen days up to now). A regimen of three drops per day of citalopram, up to seven drops in the next ten days (starting dose: three drops per day for two days, then one more drop per day every two days) and five drops of clonazepam ^61^ in the evening for two months (then gradually reduced until suspension: reduced to three drops in the evening for one week and then suspended) was also prescribed.

The rationale behind the decision to change our intervention was primarily based on medical and psychological history. However, carbamazepine was maintained during treatment because it was deemed to be effective in a clinical case of TN.

*Follow-up and outcomes*

The patient reported pain (SF-MPQ: score 1) and mood symptom relief with a subjective perception of a satisfactory quality of life. After drug prescription, the patient was followed-up every 15 days and was asked about pain intensity (SF-MPQ: score from 1 to 0) and characteristics and psychological suffering.

After two months, he reported decisive improvements in symptoms (SF-MPQ: score 1-0) and psychological suffering. Therefore, he was prescribed to gradually reduce antianxiety drug doses until suspension. Three months later, after he had confirmed decisive ameliorations in pain intensity (SF-MPQ: score 1-0) and features and in his psychological suffering, the dosage of antidepressant drug (citalopram) was reduced from seven to five drops per day. The regimen of carbamazepine was maintained (200 mg three times daily).

The patient has reported mild symptomatology since then, but his uneasiness during pain attacks has manifested as fear of pain recurrence: for these reasons, the patient is now in a 15-day follow-up program with the psychiatrist and is also currently under a regimen of five drops of citalopram per day and is still adherent to a reduced dosage of 200 mg of carbamazepine twice a day. ^61^ Subsequently, we also included the patient in a six month-recall program to accomplish dental hygiene.

Phone calls, e-mails and phone text messages, besides visits, were efficient tools to assess patient prescription adherence and tolerability. We report no adverse or unanticipated events in the described clinical case.

When facing a patient with comorbid psychological disorders associated with neuropathic pain, the dental team should preserve patient confidence, reduce anxiety and depression and promote compliance. In addition to conventional therapies, the dentist should be prepared to supplement a behavioral approach, a psychiatric/psychological consultation and a pharmacological treatment to adopt appropriate patient-health-centered medical care, modulated and balanced based on patient needs. Remarkably, a multidisciplinary dental team encompassing psychiatrists and clinical nurse specialists is key for providing personalized care to each patient. ^64^

## Discussion

Orofacial pain is always demanding and challenging—and thus often stimulating—for the practitioner. Long-lasting pain occurring before treatment can represent a risk factor for chronicization, ^3^ which in turn makes the treatment troublesome. ^2^ This implies that a prompt and accurate differential diagnosis is key for an effective treatment. However, the diagnostic process might be particularly difficult when neuropathic non-odontogenic pain is reported. ^1^ ^,^ ^2^ Medical diagnosis can vary tremendously if a patient with pain below the imaginary line drawn across the eyes is assessed by a dentist or another medical specialist. While orofacial pain is probably attributed to dental pathology in a dental environment, different diagnoses can be proposed by other medical doctors, such as neurologists and maxillo-facial surgeons. ^16^ For the diagnostic process, a comprehensive record of pain history as well as an extraoral and intraoral examination of the head and neck regions is mandatory. Furthermore, laboratory investigations and imaging techniques can be helpful. ^16^

Misleading diagnoses can lead the pain to become persistent and enhanced in its degree and characteristics, ^2^ which in turn may cause stress to the patient and clinician. A lack of knowledge, diagnostic procrastination and possibly useless irreversible dental treatments can lead to frustration ^5^ and even more severe psychological distress as a consequential comorbidity, which will be harder to manage. Dealing with a wide range of patients, from the relaxed and collaborative to anxious and depressed individuals, the dental team should have a patient-health-centered approach, optimizing and tailoring the treatment considering the patients’ psychological profiles, pathologies and pain characteristics. Therefore, a comprehensive anamnesis, including psychological assessment and history listening, is necessary. ^3^ ^,^ ^16^ Drug history should also be included. ^16^

The AO and TN cases reported well demonstrate that the psychological, behavioral and psychopharmacological approach adopted was effective in controlling pain intensity. An accurate differential diagnostic process was the key to draw a combination of pharmacological and psychological treatments. This approach drastically reduced pain duration over time, ameliorating the clinical scenario and improving patient satisfaction. However, the evaluation of the comorbid psychiatric disorders was not based on a standardized scale or a questionnaire, because these are not usually available in a dental office. A better-defined approach should be envisaged so that such instruments can become widespread in dental practices, as this would be desirable.

We concluded that an interdisciplinary approach is mandatory for the diagnosis and management ^65^ of complex pathophysiological conditions and comorbid psychological disorders. ^64^ In general, we support the notion that a first important step for the patient is that the pain is acknowledged by the clinician as real. ^16^ Psychiatric counselling and cognitive behavioral therapies, along with a specific psychopharmacological approach, can be effective treatments for patients suffering from acute anxiety, distress and depression while experiencing neuropathic pain. ^62^ The advantage is that the patient may become more compliant, and even major pain will be kept under control, with a reduced symptomatology duration. Therefore, the dental office should have a sound professional collaboration with a multidisciplinary team, including specialist psychiatrists and clinical nurse specialists. ^64^

Dentists need to be well trained in the specific field of orofacial pain to avoid diagnostic delays and multiple irreversible and ineffective dental treatments. Differential diagnosis may be difficult and challenging, especially when the pain is non-odontogenic. Dental practitioners must be familiar with the signs and symptoms related to these conditions. Moreover, they should consider that orofacial pain can often lead to comorbid psychological disorders, because its effects on the emotional state may modulate pain perception. Psychiatric and/or psychological counselling and proper drug management, together with an empathic attitude, might be crucial factors for patient compliance and improvement in clinical conditions. In the differential diagnostic process, it is imperative that dentists refrain from focusing on ordinary and common sources of tooth pain and adopt a comprehensive patient-health-centered approach to avoid the risk of aggravating patients’ clinical conditions.

## Data availability

All data underlying the results are available as part of the article and no additional source data are required.

## Consent

Written informed consent for publication of their clinical details was obtained from the patients.

## Footnotes

^1^
The classification proposed in the literature is heterogeneous, but, in accordance with the third edition of the international classification of headache disorders (ICHD-3), this condition can be classified as a subtype of persistent idiopathic facial pain, or due to occasional presence of traumatic trigger, may also be considered a subdivision of post-traumatic painful trigeminal neuropathy. ^24^
