# Supplementary material for: Atypical odontalgia and trigeminal neuralgia: psychological, behavioral and psychopharmacological approach in a dental clinic – an overview of pathologies related to the challenging differential diagnosis in orofacial pain
Source: F1000Res. 2022 Jun 27;10:317. Originally published 2021 Apr 23. [Version 3] doi: 10.12688/f1000research.51845.3 (PMC9345266; doi:10.12688/f1000research.51845.3)
Supplement: Peer review history

# Peer review: Reviewer response for version 3

**DOI:** 10.5256/f1000research.135396.r142449

**Published:** 2022-08-01

**Authors:** Claudia Sommer

**Affiliations:** 1 Department of Neurology, University of Würzburg, Würzburg, Germany

The changes made are sufficient, I approve now.

Are enough details provided of any physical examination and diagnostic tests, treatment given and outcomes?

Yes

Is the background of the cases’ history and progression described in sufficient detail?

Partly

Is sufficient discussion included of the importance of the findings and their relevance to future understanding of disease processes, diagnosis or treatment?

Partly

Is the conclusion balanced and justified on the basis of the findings?

Partly

Reviewer Expertise:

Neurology, pain

I confirm that I have read this submission and believe that I have an appropriate level of expertise to confirm that it is of an acceptable scientific standard.

# Response

**Published:** 2022-09-02

**Authors:** Riccardo Tizzoni

**Affiliations:** Private Practice, Italy

Dear Prof. Claudia Sommer,

 We thank you very much indeed for the entire reviewing process of our article. We took into great consideration your valuable suggestions to amend the article.

 Thank you very much,

 I send my Best Regards

 Riccardo Tizzoni

# Peer review: Reviewer response for version 3

**DOI:** 10.5256/f1000research.135396.r142451

**Published:** 2022-07-26

**Authors:** Joanna M. Zakrzewska

**Affiliations:** 1 UCLH NHS Foundation Trust, UCL/UCLH NIHR Biomedical Research Centre, London, UK

The paper shows a lack of structure especially when describing the differential diagnosis. The cases are too long. The first case has no details of social history and how this can impact on psychological well being which were not measured using any recognised measures. The second case is very unusual and in these patients psychological factors are often related to intensity of pain. I would be concerned that readers would start prescribing liquid medications such as clonazepam (highly addictive) and citalopram rather than using other anticonvulsants as recommended by international guidelines. 

 It is important to introduce the new nomenclature rather than use the old term of atypical odontalgia. 

 For more thoughts please see here.

Are enough details provided of any physical examination and diagnostic tests, treatment given and outcomes?

No

Is the background of the cases’ history and progression described in sufficient detail?

Partly

Is sufficient discussion included of the importance of the findings and their relevance to future understanding of disease processes, diagnosis or treatment?

Partly

Is the conclusion balanced and justified on the basis of the findings?

Partly

Reviewer Expertise:

NA

I confirm that I have read this submission and believe that I have an appropriate level of expertise to state that I do not consider it to be of an acceptable scientific standard, for reasons outlined above.

# Peer review: Reviewer response for version 2

**DOI:** 10.5256/f1000research.77676.r119329

**Published:** 2022-01-11

**Authors:** Claudia Sommer

**Affiliations:** 1 Department of Neurology, University of Würzburg, Würzburg, Germany

Dear authors,

 If this article is meant for dentists, please make it clear.

 If it is for the general public, it needs to be rewritten. Then, the dentist part would be much too detailed and technical.

 The message is not clear to me:

 You show two interesting cases, but it is very difficult to generalize from these cases. So what do we learn from them?

 You do not give a comprehensive review of orofacial pain. This may not be your aim, but what is your aim?

 Before this manuscript goes back to any reviewer, it needs to be reviewed by a psychiatrist and by a neurologist for correct terminology, and by a native English speaker for language. I made many comments in the manuscript, but there is a lot more to do!

Are enough details provided of any physical examination and diagnostic tests, treatment given and outcomes?

Yes

Is the background of the cases’ history and progression described in sufficient detail?

Partly

Is sufficient discussion included of the importance of the findings and their relevance to future understanding of disease processes, diagnosis or treatment?

Partly

Is the conclusion balanced and justified on the basis of the findings?

Partly

Reviewer Expertise:

Neurology, pain

I confirm that I have read this submission and believe that I have an appropriate level of expertise to state that I do not consider it to be of an acceptable scientific standard, for reasons outlined above.

# Response

**Published:** 2022-06-22

**Authors:** Riccardo Tizzoni

**Affiliations:** Private Practice, Italy

**Reviewer #3**

**Dear authors,**

**If this article is meant for dentists, please make it clear. If it is for the general public, it needs to be rewritten. Then, the dentist part would be much too detailed and technical.**

 We thank the reviewer for the comment and we agree that it was not clear which audience the article was directed to. In turn, we substantially modified the introduction, clearly stating that our paper is meant for dentists.

**The message is not clear to me:**

**You show two interesting cases, but it is very difficult to generalize from these cases. So what do we learn from them?**

 We thank the reviewer for acknowledging our work as an interesting contribution. We also believe that the two case reports are of interest, because they stress difficulties that dental practitioners may face in the differential diagnostic process, highlight how pain chronicity can lead to comorbidity due to psychological disorders and show how these conditions may be cured. We believe that the message is now much clearer, because we extensively stated our aims in the introduction, making the following content of the paper in line with the information provided. In the Discussion, we further resumed the main points of our contribution.  

**You do not give a comprehensive review of orofacial pain. This may not be your aim, but what is your aim?**

 We appreciated the reviewer’s comment and agreed that the manuscript had to be improved in the sections highlighted. We added a paragraph within the introduction, where we clearly stated the objectives of the article. A comprehensive review of orofacial pathologies was not our main goal. Rather, we wanted to provide a synthetic guide which may be useful for dentists in the differential diagnosis.

 “ *Despite available data on single forms of orofacial pain, different pain types arising from regions of the face and mouth remain understudied. Such information would enable the familiarization of dental practitioners with the signs and symptoms of orofacial pain, especially those with non-odontogenic origins14. Therefore, our first aim was to provide an overview of the most common orofacial pathologies which dental practitioners may encounter during their activity. This overview is not meant to be a comprehensive and fully exhaustive description of each pathology. Rather, the information we provide may offer a synthetic guide to support the differential diagnostic process, which is often complex, especially when assessing non-odontogenic pain. In our description, we specifically focus on two conditions, atypical odontalgia (AO) and trigeminal neuralgia (TN), which are the most diagnosed neuropathic pains in dental practice.*

*We discuss that a prompt and correct assessment of the pathology can help prevent the development of chronic conditions and, in turn, psychological disorders. Supporting this, we describe two case reports dealing with AO and TN. Our cases well demonstrate that late diagnosis can lead to comorbidities. In these occasions, psychological treatment may be key for the successful healing of chronic neuropathies. In line with our discussion, we stress the importance of a new patient-health-centered approach, which is required during a differential diagnosis to properly identify pathologies, thus preventing the development of chronic pains and possible comorbidities due to psychological disorders*”.  

**Before this manuscript goes back to any reviewer, it needs to be reviewed by a psychiatrist and by a neurologist for correct terminology, and by a native English speaker for language. I made many comments in the manuscript, but there is a lot more to do!**

 We acknowledge the reviewer for the punctual revision to the text. We have changed the contents accordingly. Moreover, we have extensively revised the sentences.

# Peer review: Reviewer response for version 2

**DOI:** 10.5256/f1000research.77676.r97184

**Published:** 2021-10-27

**Authors:** Joanna M. Zakrzewska

**Affiliations:** 1 UCLH NHS Foundation Trust, UCL/UCLH NIHR Biomedical Research Centre, London, UK

Although some changes have been made I think these have not been radical enough. The paper remains long winded and difficult to read. There is much too technical detail in the first case. Some of the terminology used is unprofessional. and this could just reflect that English is not the first language of the authors. I attach some examples made as notes on the manuscript.

Are enough details provided of any physical examination and diagnostic tests, treatment given and outcomes?

No

Is the background of the cases’ history and progression described in sufficient detail?

Partly

Is sufficient discussion included of the importance of the findings and their relevance to future understanding of disease processes, diagnosis or treatment?

Partly

Is the conclusion balanced and justified on the basis of the findings?

Partly

Reviewer Expertise:

Expert in facial pain and report to be the world expert in trigeminal neuralgia by expertscape

I confirm that I have read this submission and believe that I have an appropriate level of expertise to state that I do not consider it to be of an acceptable scientific standard, for reasons outlined above.

# Response

**Published:** 2022-06-22

**Authors:** Riccardo Tizzoni

**Affiliations:** Private Practice, Italy

**Reviewer #1**

*Rev.1*

**This a case report article highlighting two cases of non-dental facial pain which had psychiatric co-morbidity.**

**It is now well established that all chronic conditions and especially chronic pain results in mental health co-morbidities. These should always be explored and seeing these patients in a multidisciplinary team setting is advantageous.**

**The introduction should provide the criteria of depression and anxiety as provided by the DSM classification. The IMMPACT ^1^ group have suggested the outcome measures that should be used to ascertain various aspects of pain including mood and quality of life.**

 We thank the reviewer for the comment. We have included in the introduction section a dedicated paragraph in which we define both anxiety and depression, discussing differences between these two disorders. In our study, we measured pain intensity according to the short-form McGill pain questionnaire.

**The diagnostic criteria for the two conditions could be summarized in a table using the ICHD ^2^ classification which would then highlight the similarities and differences between the two conditions.**

 We agree that a more schematic definition of the diagnostic criteria for AO and TN should have been included. We now present two separate tables describing diagnostic criteria for these two conditions. 

**The two cases should provide more detailed history rather then very detailed treatment plans. For the trigeminal neuralgia (TN) case the reader should be provided with details of onset of the condition e.g memorable, timing of each attack, severity, frequency of attacks, provoking factors, possible remission periods. According to guidelines for TN an MRI should have been done and its result reported.**

 We thank the reviewer for the comment. We have modified the description of the case reports including more detailed information according to reviewer’s comment. We have reported about trigger zones, remission period and available MRI data in the description of the patient with trigeminal neuralgia.

**How was the impact on mood and quality of life measured? SF-MPQ is insufficient and we have shown the considerable impact this condition has on life ^3^ and how this can be improved over time ^4^.**

 We thank the reviewer for the comment. We agree that our approach is limited and we clearly state this in our discussion (“ *However, the evaluation of the comorbid psychiatric disorders was not based on a standardized scale or a questionnaire, because these are not usually available in a dental office. A better-defined approach should be envisaged so that such instruments can become widespread in dental practices, as this would be desirable.*”). Even though we auspicate that a more structured approach may develop, our approach is based on common resources available to dentists. We hope that our contribution may be stimulating for possible methodology ameliorations.   

**If the pain does not respond to carbamazepine initially then the diagnosis needs to be re-visited and potentially other anti-epileptics used as per guidelines ^5^. Why use clonazepam, potentially addictive drug when addressing the TN pain which often resolves the depression as we have shown?** ^**3**^

 Oxcarbazepine has been cited with carbamazepine as first line drugs. Second line drugs have also been mentioned. In support to our decision, we reported the reference to the paper by Sindrup & Jensen (2002): Sindrup SH, Jensen TS. Pharmacotherapy of trigeminal neuralgia. Clin J Pain. 2002 Jan-Feb;18(1):22-7. doi: 10.1097/00002508-200201000-00004. PMID: 11803299.

**These patients certainly benefit from a cognitive behaviour programme ^6^. A liaison psychiatrist is a useful addition to the team but one needs clinical psychologists and clinical nurse specialists as we have shown in our pathway ^7^.**

 We acknowledge the reviewer for these tips. We have included a brief reference to these publications within the description of our TN case report:

 “ *Cognitive behavioral therapy has been demonstrated to be an effective approach to improve life quality of TN patients ^6^*”.

 “ *Remarkably, a multidisciplinary dental team encompassing psychiatrists and clinical nurse specialists would be the key for providing personalized care to each patient ^65^*”.

 “ *We concluded that an interdisciplinary approach is mandatory for diagnosis and management ^58 ^of complex pathophysiological conditions and comorbid psychological disorders ^66^*”.

 “ *Psychiatric counselling and cognitive behavioural therapies, along with a specific psychopharmacologic approach, can be effective treatments for patients suffering from acute anxiety, distress and depression while experiencing neuropathic pain ^64^*”.

 “ *The dental office, therefore, should have a good, continuing professional collaboration with a multidisciplinary team, including specialist psychiatrists and clinical nurse specialists ^65^*”.

**Discussion is very long winded and makes an attempt to provide differential diagnosis. This could best be summarised through a table as has been done in other publications.**

 We agree and substantially modified the discussion. Description of orofacial pathologies was moved to the Introduction. The Discussion is now more compact, focusing on the key information that we wanted do highlight with our contribution.

**I do not think this article provides any new information. There is an excellent series of articles on facial pain in a special issue of Cephalalgia 2017 Vol 37 No 7.**

 We are sorry to read that the reviewer does not think that the manuscript may represent a positive contribution. Even if we agree that many excellent papers are available on facial pain, we also believe that our article can be of help for many dental practitioners to reach an effective differential diagnosis. Also, our case reports can represent two interesting practical examples for the treatment of AO and TN.

*Rev. 2*

**Although some changes have been made I think these have not been radical enough. The paper remains long winded and difficult to read. There is much too technical detail in the first case. Some of the terminology used is unprofessional. and this could just reflect that English is not the first language of the authors. I attach some examples made as notes on the manuscript.**

 We thank the reviewer for the new comments to the manuscript. We agree that the content was sometimes too long winded. For this reason, we have radically restructured the text. Although preserving the information provided in the first version of the manuscript, paragraphs have been reorganized to favor the reading process. In the introduction, we now provide the rationale behind our contribution, we clearly state our aims and finally offer a synthetic overview of orofacial neuropathies. When describing our case reports, we believe that details that we provided offer an interesting contribution to thoroughly show the process that led us to the differential diagnosis and how we treated the pathology. Even if case report description is still wide, we also think that the information included will be of interest to the readers and can improve our contribution.

 We agree with the reviewer that the style had to be revised. Our revision process encompassed a substantial modification of the periods to make the content more professional and easier to read.

 We believe that the manuscript improved a lot since the last version that we presented and we think that it can be now considered available for publication. Most importantly, we also believe that our paper could represent a positive contribution to dental practitioners.

# Peer review: Reviewer response for version 2

**DOI:** 10.5256/f1000research.77676.r97183

**Published:** 2021-10-25

**Authors:** Giulia Di Stefano

**Affiliations:** 1 Department of Human Neuroscience, Sapienza University, Rome, Italy

The authors addressed all issues. I have no further comments

Are enough details provided of any physical examination and diagnostic tests, treatment given and outcomes?

Partly

Is the background of the cases’ history and progression described in sufficient detail?

Yes

Is sufficient discussion included of the importance of the findings and their relevance to future understanding of disease processes, diagnosis or treatment?

Partly

Is the conclusion balanced and justified on the basis of the findings?

Partly

Reviewer Expertise:

Facial Pain, Neuropathic pain mechanisms, treatment of trigeminal neuralgia

I confirm that I have read this submission and believe that I have an appropriate level of expertise to confirm that it is of an acceptable scientific standard.

# Response

**Published:** 2021-10-25

**Authors:** Riccardo Tizzoni

**Affiliations:** Private Practice, Italy

Dear Prof. Di Stefano we thank you very very much indeed.

 Your reviewing process stimulated us to ameliorate the manuscript, amending it following your precious suggestions.

 Thank you!

 We send you our Best Regards.

 Riccardo Tizzoni, Marta Tizzoni and Carlo Alfredo Clerici

# Peer review: Reviewer response for version 1

**DOI:** 10.5256/f1000research.55055.r90507

**Published:** 2021-08-12

**Authors:** Giulia Di Stefano

**Affiliations:** 1 Department of Human Neuroscience, Sapienza University, Rome, Italy

In this paper, the Authors described two cases of orofacial pain, focusing on the psychiatric comorbidity and the need of a multidisciplinary treatment approach.

 I have the following suggestions to improve the quality of the report.

- Diagnostic criteria should be reported more clearly in a table.
- In patients with odontalgia, the neuropathic origin of pain should be excluded through neurophysiological tests (trigeminal reflexes) evaluating trigeminal afferents integrity. I suggest mentioning this point in the text.
- I suggest citing the etiological classification of trigeminal neuralgia. Recent diagnostic criteria distinguish trigeminal neuralgia as “classical”, related to neurovascular compression producing morphological changes on the trigeminal root, ‘‘secondary’’ to a major neurological disease or ‘‘idiopathic’’ with unknown etiology. Genetic factors may play a role in the pathophysiology of idiopathic trigeminal neuralgia. These points should be addressed in the text
- Pathophysiology of paroxysmal pain should be expanded. Vascular compression is the usual cause of demyelination at the site just before the nerve enters the pons, and multiple sclerosis is the typical cause at the site just after entry into the pons.
- The possibility of concomitant continuous in a significant portion of patients with trigeminal neuralgia should be cited.
- In the description of patients with trigeminal neuralgia, the term “pain amplitude” should be replaced with “pain intensity”
- Were trigger zones or maneuvers reported by the patient? What about remission periods? Were MRI data available?
- In the discussion, I suggest a more concise description of the alternative orofacial pain diagnosis. Some sections seem redundant.  
- Oxcarbazepine should be cited with carbamazepine as first line drugs. Second line drugs should be mentioned.

Are enough details provided of any physical examination and diagnostic tests, treatment given and outcomes?

Partly

Is the background of the cases’ history and progression described in sufficient detail?

Yes

Is sufficient discussion included of the importance of the findings and their relevance to future understanding of disease processes, diagnosis or treatment?

Partly

Is the conclusion balanced and justified on the basis of the findings?

Partly

Reviewer Expertise:

Facial Pain, Neuropathic pain mechanisms, treatment of trigeminal neuralgia

I confirm that I have read this submission and believe that I have an appropriate level of expertise to confirm that it is of an acceptable scientific standard, however I have significant reservations, as outlined above.

# Response

**Published:** 2021-09-26

**Authors:** Riccardo Tizzoni

**Affiliations:** Private Practice, Italy

Dear Prof. Giulia Di Stefano we thank you very much for the entire, precious process of reviewing our article. The comments and suggestions indicated by you have been considered and we hope satisfactorily addressed.

 We amended the whole manuscript accordingly.

 Thank you very much.

 I send you my Best Regards

 Riccardo Tizzoni

# Peer review: Reviewer response for version 1

**DOI:** 10.5256/f1000research.55055.r83851

**Published:** 2021-05-14

**Authors:** Joanna M. Zakrzewska

**Affiliations:** 1 UCLH NHS Foundation Trust, UCL/UCLH NIHR Biomedical Research Centre, London, UK

This a case report article highlighting two cases of non dental facial pain which had psychiatric co-morbidity.

 It is now well established that all chronic conditions and especially chronic pain results in mental health co-morbidities. These should always be explored and seeing these patients in a multidisciplinary team setting is advantageous.

 The introduction should provide the criteria of depression and anxiety as provided by the DSM classification. The IMMPACT ^1^ group have suggested the outcome measures that should be used to ascertain various aspects of pain including mood and quality of life.

 The diagnostic criteria for the two conditions could be summarized in a table using the ICHD ^2^ classification which would then highlight the similarities and differences between the two conditions.

 The two cases should provide more detailed history rather then very detailed treatment plans. For the trigeminal neuralgia (TN) case the reader should be provided with details of onset of the condition e.g memorable, timing of each attack, severity, frequency of attacks, provoking factors, possible remission periods. According to guidelines for TN an MRI should have been done and its result reported. How was the impact on mood and quality of life measured? SF-MPQ is insufficient and we have shown the considerable impact this condition has on life ^3^ and how this can be improved over time ^4^. If the pain does not respond to carbamazepine initially then the diagnosis needs to be re-visited and potentially other anti-epileptics used as per guidelines ^5^. Why use clonazepam, potentially addictive drug when addressing the TN pain which often resolves the depression as we have shown? ^3^.

 These patients certainly benefit from a cognitive behaviour programme ^6^.

 A liaison psychiatrist is a useful addition to the team but one needs clinical psychologists and clinical nurse specialists as we have shown in our pathway ^7^.

 Discussion is very long winded and makes an attempt to provide differential diagnosis. This could best be summarised through a table as has been done in other publications.

 I do not think this article provides any new information. There is an excellent series of articles on facial pain in a special issue of Cephalalgia 2017 Vol 37 No 7.

Are enough details provided of any physical examination and diagnostic tests, treatment given and outcomes?

No

Is the background of the cases’ history and progression described in sufficient detail?

Partly

Is sufficient discussion included of the importance of the findings and their relevance to future understanding of disease processes, diagnosis or treatment?

Partly

Is the conclusion balanced and justified on the basis of the findings?

Partly

Reviewer Expertise:

Expert in facial pain and report to be the world expert in trigeminal neuralgia by expertscape

I confirm that I have read this submission and believe that I have an appropriate level of expertise to state that I do not consider it to be of an acceptable scientific standard, for reasons outlined above.

## References

[1] : Core outcome measures for chronic pain clinical trials: IMMPACT recommendations. *Pain* .2005;113(1-2) : 10.1016/j.pain.2004.09.012 9-19 10.1016/j.pain.2004.09.012 15621359

[2] Headache Classification Committee of the International Headache Society (IHS) The International Classification of Headache Disorders, 3rd edition. *Cephalalgia* .38(1) : 10.1177/0333102417738202 1-211 10.1177/0333102417738202 29368949

[3] : Evaluating the impact of trigeminal neuralgia. *Pain* .158(6) : 10.1097/j.pain.0000000000000853 1166-1174 10.1097/j.pain.0000000000000853 28114183

[4] : Burden of illness of trigeminal neuralgia among patients managed in a specialist center in England. *J Headache Pain* .2020;21(1) : 10.1186/s10194-020-01198-z 130 10.1186/s10194-020-01198-z 33167869PMC7653862

[5] : European Academy of Neurology guideline on trigeminal neuralgia. *Eur J Neurol* .26(6) : 10.1111/ene.13950 831-849 10.1111/ene.13950 30860637

[6] : Cognitive Behavioral Therapy for Patients with Trigeminal Neuralgia: A Feasibility Study. *Journal of Oral & Facial Pain and Headache* .2021;35(1) : 10.11607/ofph.2664 30-34 10.11607/ofph.2664 33730124

[7] : Trigeminal neuralgia and its variants. Management and diagnosis. *Oral Surgery* .2020;13(4) : 10.1111/ors.12464 404-414 10.1111/ors.12464

# Response

**Published:** 2021-09-26

**Authors:** Riccardo Tizzoni

**Affiliations:** Private Practice, Italy

Dear Prof. Joanna M. Zakrzewska, we thank you very much for the reviewing process.

 Your review stimulated us to amend and ameliorate the article, creating a second version.

 We hope that our responses and revisions may now satisfy you.

 Again, thank you very much

 I send my Best Regards,

 Riccardo Tizzoni
